# Mass Spectrometry-Based Proteomic Characterization of Cutaneous Melanoma Ectosomes Reveals the Presence of Cancer-Related Molecules

**DOI:** 10.3390/ijms21082934

**Published:** 2020-04-22

**Authors:** Magdalena Surman, Sylwia Kędracka-Krok, Dorota Hoja-Łukowicz, Urszula Jankowska, Anna Drożdż, Ewa Ł. Stępień, Małgorzata Przybyło

**Affiliations:** 1 Department of Glycoconjugate Biochemistry, Institute of Zoology and Biomedical Research, Faculty of Biology, Jagiellonian University, 30-387 Kraków, Poland; magdalena.surman@doctoral.uj.edu.pl (M.S.); dorota.hoja-lukowicz@uj.edu.pl (D.H.-Ł.); 2 Department of Physical Biochemistry, Faculty of Biochemistry, Biophysics and Biotechnology, Jagiellonian University, 30-387 Kraków, Poland; sylwia.kedracka-krok@uj.edu.pl; 3 Proteomics and Mass Spectrometry Core Facility, Malopolska Centre of Biotechnology, Jagiellonian University, 30-387 Kraków, Poland; urszula.jankowska@uj.edu.pl; 4 Department of Medical Physics, M. Smoluchowski Institute of Physics, Faculty of Physics, Astronomy and Applied Computer Science, Jagiellonian University, 30-348 Kraków, Poland; anna.drozdz@uj.edu.pl (A.D.); e.stepien@uj.edu.pl (E.Ł.S.)

**Keywords:** biomarkers, cutaneous melanoma, ectosomes, extracellular vesicles, invasion, metastasis, proteomics

## Abstract

Cutaneous melanoma (CM) is an aggressive type of skin cancer for which effective biomarkers are still needed. Recently, the protein content of extracellular vesicles (ectosomes and exosomes) became increasingly investigated in terms of its functional role in CM and as a source of novel biomarkers; however, the data concerning the proteome of CM-derived ectosomes is very limited. We used the shotgun nanoLC–MS/MS approach to the profile protein content of ectosomes from primary (WM115, WM793) and metastatic (WM266-4, WM1205Lu) CM cell lines. Additionally, the effect exerted by CM ectosomes on recipient cells was assessed in terms of cell proliferation (Alamar Blue assay) and migratory properties (wound healing assay). All cell lines secreted heterogeneous populations of ectosomes enriched in the common set of proteins. A total of 1507 unique proteins were identified, with many of them involved in cancer cell proliferation, migration, escape from apoptosis, epithelial–mesenchymal transition and angiogenesis. Isolated ectosomes increased proliferation and motility of recipient cells, likely due to the ectosomal transfer of different cancer-promoting molecules. Taken together, these results confirm the significant role of ectosomes in several biological processes leading to CM development and progression, and might be used as a starting point for further studies exploring their diagnostic and prognostic potential.

## 1. Introduction

Cutaneous melanoma (CM) is the most aggressive type of skin cancer, and its incidence is increasing rapidly with approximately 350,000 new cases worldwide each year [1]. CM’s growing prevalence has increased the necessity for research into improved diagnostic, preventative and treatment methods; however, early detection and disease constraint still present significant challenges.

As successful isolation protocols developed, extracellular vesicles (EVs) became widely investigated in terms of their functional role in CM and as a possible source of disease biomarkers. Viable cells predominantly release two types of EVs: ectosomes, directly shed from the plasma membrane, and exosomes, released after fusion of multivesicular bodies (MVBs) with the plasma membrane. In the case of apoptotic cells, cell fragmentation and formation of apoptotic bodies, the third type of EV, containing nuclear and cytoplasmic material as well as intact organelles, is observed [2]. The specific content of EVs and their action towards recipient cells depend on their molecular composition, which is determined by the cell of their origin. Proteins, lipids and/or nucleic acids specific for a given cell type can be detected in EVs after their isolation from body fluids or conditioned media [3]. Such accessibility of EVs contributes to their prognostic and diagnostic value and indicates their potential as an alternative to invasive biopsy procedures.

Compared with extensive research on melanoma-derived exosomes [4,5], ectosomes appear as an understudied population of EVs, though their tumor-promoting activity has already been recognized. Ectosomes were shown to modulate extracellular matrix degradation during CM invasion and metastasis [6,7,8], tissue factor (TF)-related procoagulant state [9], tumor-stroma interactions [10], as well as suppression of immune response [11].

Besides functional studies, qualitative and quantitative proteomics emerged as a major tool for the identification of particular molecules within ectosomes, which account for their tumor-promoting activity. Due to the high throughput and sensitivity of mass spectrometry, the number of proteomic EV-oriented studies are constantly increasing and creating alternative analytical approaches, such as gel-based or shotgun proteomics. Novel, state-of-the-art methods allow identification of over 1000 proteins in one EV sample originating from different melanoma cell lines and solid melanoma tumors [5].

Nevertheless, the amount of data concerning the proteome of CM-derived EVs is still very limited. Considering the potential role of EVs in the development and progression of CM, the present study uses a nanoLC–MS-based proteomic approach to investigate the protein content of ectosomes derived from two primary and metastatic CM cell lines. In addition, cancer-promoting effects exerted by CM-derived ectosomes on equally or less invasive recipient cells in terms of cell proliferation and migratory properties were assessed.

## 2. Results

### 2.1. Size and Marker Characterization of CM Ectosomes

**Figure 1:**
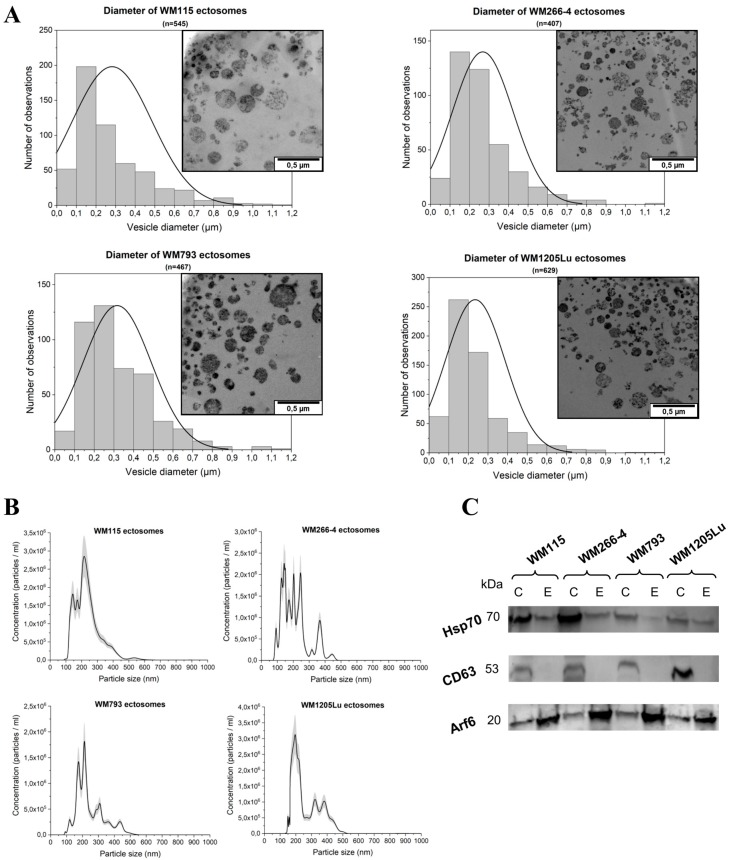
Analysis of ectosome sample purity. (**A**) TEM analysis of cutaneous melanoma (CM)-derived ectosomes. Size distributions are presented on histograms. Mean diameter ± standard deviation was calculated for all observed vesicles (n) from a given sample. (**B**) Nanoparticle Tracking Analysis (NTA) analysis of CM-derived ectosomes. Results from five independent measurements for each CM cell line are presented on graphs. The shaded area depicts standard deviation. (**C**) Western blot analysis of extracellular vesicle (EV) markers. Fifty μg of proteins from whole-cell protein extracts (lines C) and ectosome samples (lines E) separated by 10% SDS-PAGE and transferred into PVDF membrane were probed with anti-CD63 (1:2000), anti-HSP70 (1:2000) and anti-ARF6 (1:500) as primary antibodies and anti-mouse IgG-HRP (1:400) as a secondary antibody. WM115 (primary) and WM266-4 (metastatic), cell lines originating from the same individual, radial/vertical growth phase and lymph node metastasis, respectively; primary WM793 cell line, representing the vertical growth phase; WM1205Lu, a metastatic variant of WM793 cells obtained from lung metastasis.

In the present study, ectosome isolation based on sequential centrifugation with final centrifugation at 18,000× *g* was applied. Transmission Electron Microscopy (TEM), Nanoparticle Tracking Analysis (NTA) and Western Blot (WB) were performed to validate the sample purity and effectiveness of isolation. Ultrathin TEM sections of ectosome samples derived from four CM cell lines contained numerous, distinguishable vesicles (Figure 1A). The observed populations were heterogeneous and contaminated neither with cells nor with cellular organelles. Regarding particle size, only a few vesicles smaller than 100 nm or larger than 1 µm in diameter were found (Figure 1A). Ectosome samples were simultaneously analyzed by NTA (Figure 1B). The obtained results confirmed that a majority of vesicles were larger than 100 nm, proving contamination with exosomes negligible. Moreover, according to both TEM and NTA measurements, ectosomes with a diameter range of 100–300 nm constituted the most abundant subpopulation of ectosomes in each sample. Additionally, the absence or depletion of classical exosomal protein markers, CD63 and Hsp70, was demonstrated for each ectosome sample (Figure 1C). In contrast, ectosome samples were enriched in ARF6, the protein involved directly in the shedding of plasma membrane-derived EVs but not in exosome biogenesis. Based on the above evidence, we considered the isolated EV population to be highly enriched in ectosomes.

### 2.2. Identified Proteins of CM Ectosomes and Their Functional Classification

**Figure 2:**
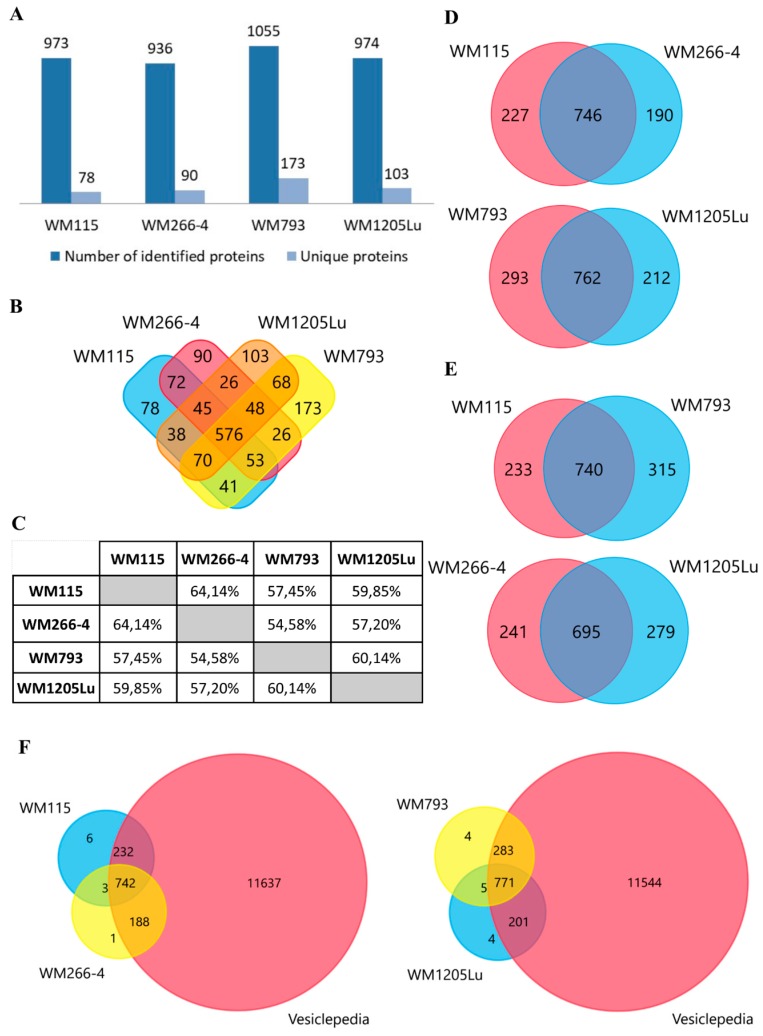
(**A**) Number of proteins identified in two biological replicates of each CM ectosome sample by at least two peptides. (**B**) Venn diagram illustrating the number of proteins shared between given ectosome samples. (**C**) Percentage of proteins shared between given ectosome samples. Venn diagrams illustrating the number of proteins shared between ectosomes released by isogenic (**D**) and primary or metastatic CM cells (**E**). Venn diagram illustrating protein overlap between CM ectosomes and Vesiclepedia database as a reference (**F**). Ectosomes were isolated from WM115 (primary) and WM266-4 (metastatic) cell lines originating from the same individual, radial/vertical growth phase and lymph node metastasis, respectively; primary WM793 cell line, representing vertical growth phase; and WM1205Lu, a metastatic variant of WM793 cells obtained from lung metastasis.

Protein profiles for ectosomes secreted by four CM cell lines were obtained using the gel-free nanoLC–MS/MS proteomic approach. For all cell lines, a total of 1507 unique proteins (listed in Supplementary Materials Data 1) were identified in two biological replicates and with at least two peptides. Regarding particular cell lines, ectosomes from metastatic WM793 cells had the highest number of 1055 proteins, while ectosomes from metastatic WM266-4 cells had the lowest number of 936 proteins (Figure 2A). In all ectosomal samples, 576 proteins were present, while the number of proteins unique for a given cell line ranged between 78 (primary WM115 cells) and 173 (primary WM793 cells). Moreover, a comparison to the Vesiclepedia database was made (Figure 2F) for each ectosome sample. The vast majority of proteins identified by the present study were also detected by other vesicle-related studies, supporting their vesicular origin instead of being a part of co-isolated cell debris, etc.

**Figure 3:**
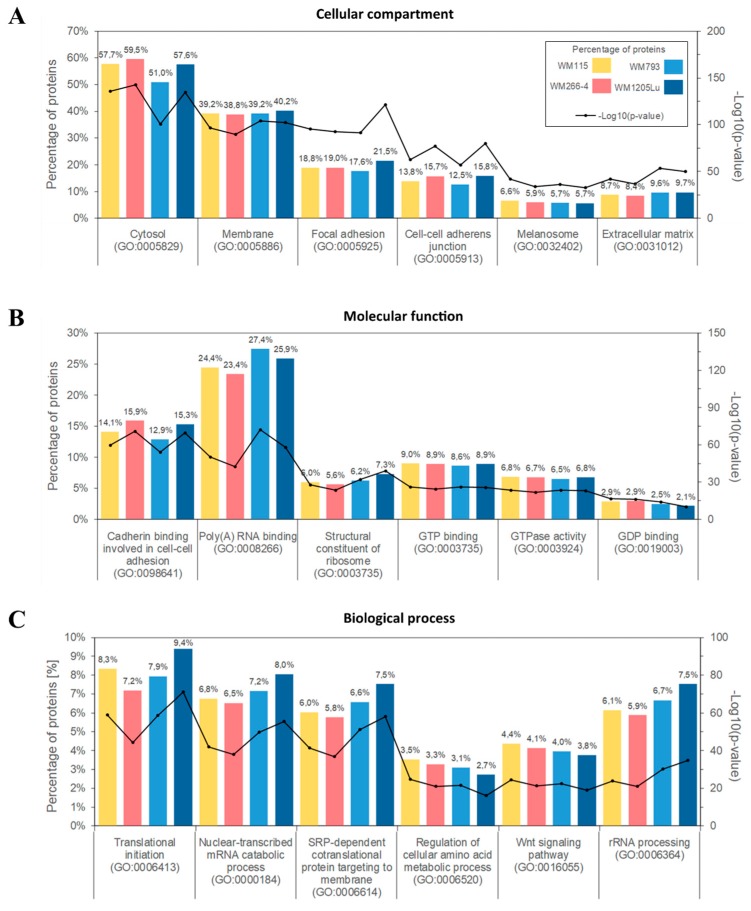
Gene ontology (GO) analysis of CM ectosomal proteins from four different cell lines performed with the use of FunRich 2.0 software with UniProt (release 2019_11) database as a reference. For each GO term (“Cellular compartment” (**A**), “Molecular function” (**B**), “Biological process” (**C**)), six categories with the highest statistical significance of protein enrichment within the specific category (*p* < 0.001) were presented on graphs. Ectosomes were isolated from WM115 (primary) and WM266-4 (metastatic) cell lines originating from the same individual, radial/vertical growth phase and lymph node metastasis, respectively; primary WM793 cell line, representing vertical growth phase; and WM1205Lu, a metastatic variant of WM793 cells obtained from lung metastasis.

Subsequently, Gene Ontology (GO) analysis was performed to group identified proteins according to the cellular compartments of their origin, molecular functions and involvement in different biological processes. For each cell line, the largest group of ectosomal proteins was associated with cytosolic (up to 59.5% of identified proteins) or membrane origin (up to 40.2%, Figure 3A). This finding reflects the mechanism of ectosome biogenesis which involves the accumulation of their components in particular domains within the membranes of origin. In support of the plasma membrane origin of ectosomes, many of the identified proteins were associated with cell–cell adhesion including adherent junctions (up to 15.8%) and focal adhesion (up to 21.5%). Moreover, approximately 6% of proteins from each ectosomal sample were related to melanosomes, reflecting the melanocytic origin of CM cells.

Regarding molecular functions (Figure 3B), the most abundant groups of proteins were related to cadherin binding during cell–cell adhesion, binding of RNA and signal transduction. Particularly, nucleotide-binding proteins were widely represented alongside proteins involved in Wnt, TNF-mediated or MAPK signaling pathways. Annotations related to biological processes (Figure 3C) revealed the enrichment in proteins involved in translation and co-translational processes such as RNA metabolism/processing and protein targeting. Strikingly, none of the aforementioned classifications showed noticeable differences between samples, suggesting that the ultimate role of the ectosomal proteome for CM cells in in vitro culture was similar for all CM cell lines.

**Figure 4:**
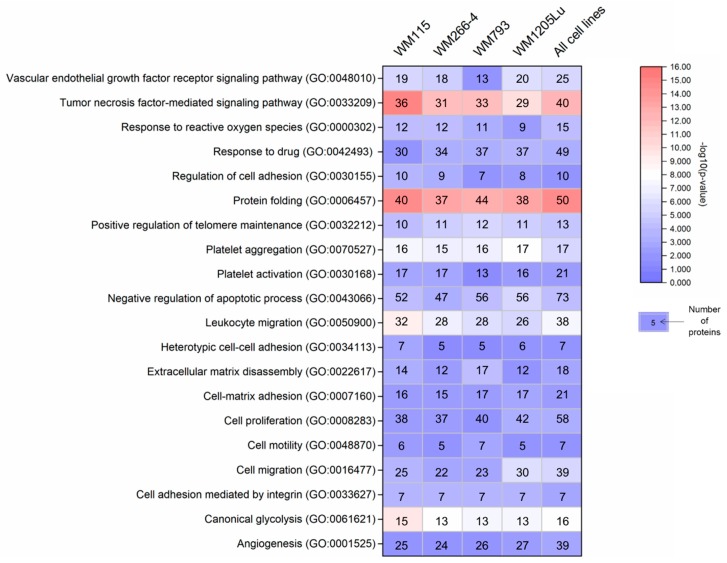
Gene ontology annotations by the biological process for each CM ectosome sample performed with the use of FunRich 2.0 software via UniProt (release 2019_11) database. Numbers of proteins within the chosen cancer-related categories (with statistical significance of protein enrichment within the given category calculated as −log10(*p* value)) are shown on the heat map. Ectosomes were isolated from WM115 (primary) and WM266-4 (metastatic) cell lines originating from the same individual, radial/vertical growth phase and lymph node metastasis, respectively; primary WM793 cell line, representing vertical growth phase; and WM1205Lu, a metastatic variant of WM793 cells obtained from lung metastasis.

**Figure 5:**
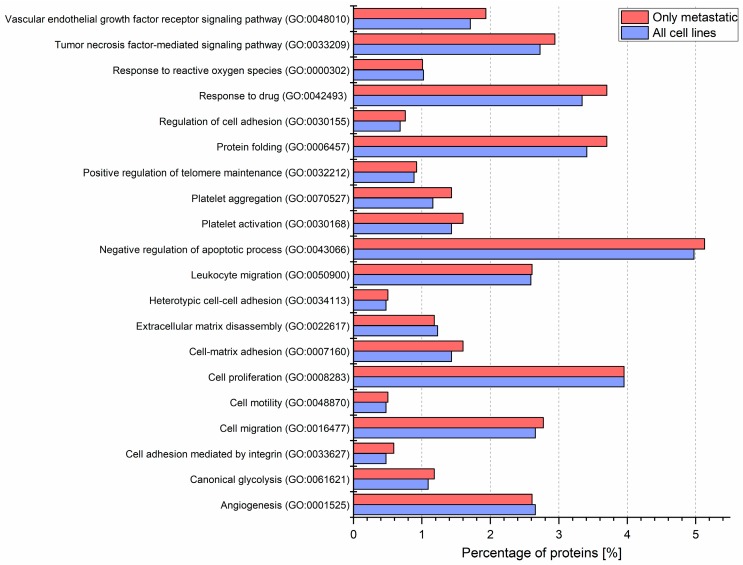
Gene ontology annotations by the biological process for each CM ectosome sample performed via UniProt (release 2019_11) database. Percentages of proteins within the chosen cancer-related categories for ectosomes from all or metastatic cell lines are presented on the graph. Ectosomes were isolated from WM115 (primary) and WM266-4 (metastatic) cell lines originating from the same individual, radial/vertical growth phase and lymph node metastasis, respectively; primary WM793 cell line, representing vertical growth phase; and WM1205Lu, a metastatic variant of WM793 cells obtained from lung metastasis.

To gain insight into how recipient cells could be impacted by CM ectosomes during cancer progression, selected cancer-related GO categories and a number of protein annotations were presented on the heat map (Figure 4). Categories with the highest numbers of annotated proteins included TNF-mediated signaling, cell proliferation, negative regulation of apoptosis, cell migration and angiogenesis, reflecting the cancer-promoting effect of CM ectosomes (Figure 4). Moreover, the percentage of annotations for analogous categories was compared between the lists of proteins from all and only metastatic CM ectosome samples (Figure 5). The percentage of proteins assigned to the majority of chosen categories was higher for ectosomes from metastatic cell lines, which corresponds to their more aggressive phenotype.

When comparing only differential proteins from WM115 and WM793 ectosomes, GO analysis indicated that ectosomes isolated from WM115 cells are better equipped with proteins involved in transport, especially the proteins related to vesicle-mediated transport (Supplementary Materials Data 2, Figure S1). Additionally, MHC class II receptor activity GO category was enriched for WM115 ectosomes when compared with ectosomes derived from WM266-4 cells (Supplementary Materials Data 2, Figure S2), which is in line with the results of comparative transcriptome studies of MHC-II expression in these cell lines [12]. The comparison between WM793 and WM1205Lu ectosomes revealed that WM793 ectosomes carry more proteins involved in immunity regulation, while WM1205Lu ectosomes comprise more proteins that regulate migration, localization and adherent junction (Supplementary Materials Data 2, Figures S3 and S4). Finally, the comparison between ectosomes obtained from two metastatic cell lines, WM266-4 and WM1205Lu, showed that WM1205Lu ectosomes contain mainly proteins of MHC-I complex, while WM266-4 ectosomes contain both MHC-I and MHC-II proteins (Supplementary Materials Data 2, Figures S5 and S6).

In summary, ectosomes obtained from two independent isogenic melanoma models (primary tumor vs. metastasis within one donor) WM115/WM266-4 and WM793/WM1205Lu cell lines were used to assess whether their cellular origin (skin or lymph node/lung metastasis) affect their overall molecular composition and function. However, regardless of their origin (corresponding to different disease stages), CM ectosomes displayed an almost identical pattern of enrichment within the most abundant GO categories, which is likely related to the common part of their proteome, i.e., 576 of shared proteins (Figure 2B). Proteins unique for CM ectosomes from given cell line do not, therefore, appear to affect basic processes and molecular functions mediated by vesicles; nevertheless, they might be responsible for their cancer-promoting effects.

### 2.3. Cancer-Promoting Effect of Ectosomes Derived from Metastatic Cells on Less Invasive Cells

**Figure 6:**
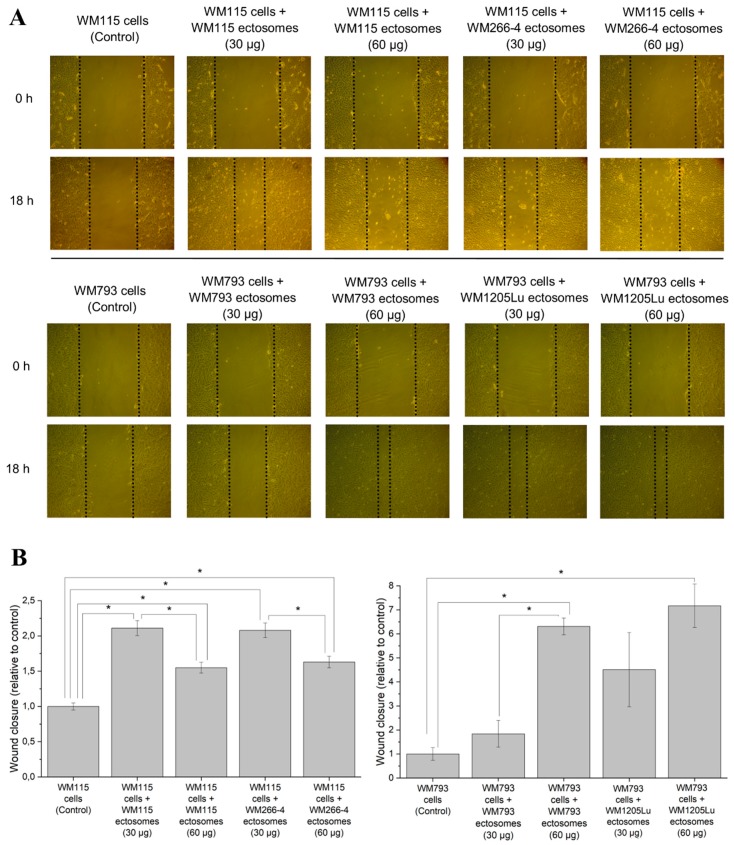
Effect exerted by self-derived ectosomes and ectosomes released by their isogenic metastatic cells, on the motility of primary WM115 and WM793 cells. Wound healing assay was performed after 18 h of incubation with ectosomes. (**A**) Representative images were taken at 0 h and at 18 h. (**B**) Graphs presenting relative velocity of wound closure calculated from three repetitions. “*” denotes statistically significant differences (Tukey’s post-hoc test, *p* value < 0.05).

In connection with the presence of multiple cancer-promoting factors demonstrated by proteomics, functional tests were performed to prove that proteins carried by ectosomes indeed modulate the function of recipient cells. It was evaluated whether self-derived ectosomes and ectosomes released by isogenic metastatic cells exert a cancer-promoting functional effect on WM115 and WM793 cells. In wound healing assay, the addition of either WM266-4- or WM1205Lu-derived ectosomes (30 µg or 60 µg of protein) increased the motility of recipient cells (Figure 6). In the case of WM115 cells, the velocity of wound closure was similarly 1.5–2.5 times higher in comparison to the control, regardless of whether the cells were treated with WM115 or WM266-4 ectosomes. When ectosomes were added to WM793 cell culture, a dose-dependent increase in wound closure was observed, with WM1205Lu ectosomes causing significantly higher response at corresponding doses.

**Figure 7:**
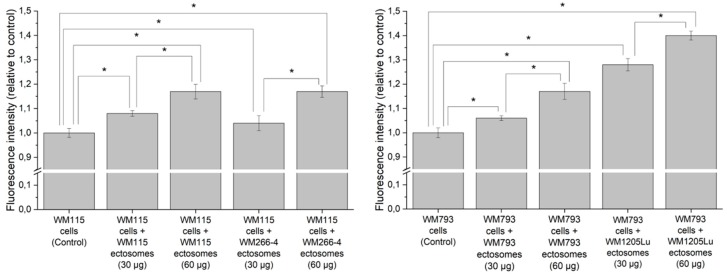
Effect of incubation with self-derived ectosomes and ectosomes released by their isogenic metastatic cells, on proliferation of primary WM115 and WM793 cells. Alamar Blue assay was carried out after 18 h of incubation with ectosomes. All experiments were conducted in triplicate. “*” denotes statistically significant differences (Tukey’s post-hoc test, *p* value < 0.05).

Additionally, Alamar Blue cell viability assay was carried out to assess ectosome-induced changes in melanoma cell proliferation rate (Figure 7). After incubation with WM266-4- and WM1205Lu-derived ectosomes (30 µg or 60 µg of protein), higher fluorescence of recipient cells was measured in comparison to the untreated control. In the case of ectosomes derived from primary CM cells, WM115 ectosomes exerted a similar effect on recipient cells as their metastatic equivalent (WM266-4 ectosomes), but WM793 ectosomes caused a lower increase in cell viability than WM1205Lu ectosomes. The observed effects were dose-dependent. To sum up, wound healing and proliferation assays proved that proteins carried by ectosomes derived from CM cells indeed modulated the function of recipient cells.

## 3. Discussion

### 3.1. Proteins Involved in Cancer Progression Detected in CM-derived Ectosomes by LC–MS/MS

In the present study, ectosomes were isolated from conditioned media of in vitro cultured four CM cell lines representing different stages of the disease. Sequential centrifugation with the final step at 18,000× *g* was applied to pellet ectosomes. The range between 16,000 and 20,000× *g* is considered to be sufficient for successful isolation of ectosomes, but insufficient for pelleting exosomes (at least approx. 100,000× *g* is required). Similar centrifugal forces can also be applied to pellet apoptotic bodies; however, viable melanoma cells in culture should not release apoptotic bodies if apoptotic processes are not over-induced [13]. Since sequential centrifugation is based on the differences in the size of isolated particles, it is biased by the size overlap between EV subpopulations. Therefore, isolated EV populations should rather be analyzed in terms of their relative depletion or enrichment. Herein, we showed, using three independent methods, i.e., TEM, NTA and Western Blot, that isolated EV samples were highly enriched in ectosomes. The majority of isolated vesicles were within the predefined size range for ectosomes, depleted of exosomal markers (CD63 and Hsp70), and enriched in ARF6, a protein marker confirming plasma membrane origin of EVs.

Obtained ectosome samples from primary (WM115, WM793) and metastatic (WM266-4, WM1205Lu) CM cell lines were next analyzed using the shotgun nanoLC–MS/MS approach to profile their protein content. GO analysis for CM ectosomes revealed the presence of proteins involved in cell proliferation, migration, escape from apoptosis, angiogenesis, etc., and most proteins belonging to these groups were more abundant in ectosomes released by metastatic CM cell lines. Moreover, the functional tests performed in the present study have shown that ectosomes stimulated proliferation and migratory properties of recipient melanoma cells, most likely due to the ectosomal transfer of different cancer-promoting molecules.

Regarding the altered adhesion and motility of CM cells, the action of integrins and cadherins has been widely studied. Increased expression of integrin αvβ3 correlated with the progression of melanoma from radial to vertical growth phase [14], while expression of integrins α2β1 and α3β1 was increased in metastatic cells compared to primary ones [15,16]. Additionally, the restored expression of E-cadherin was found to inhibit melanoma cell invasion by decreasing the expression of β3 integrins and MUC18 receptor [17]. In the present study, all the aforementioned integrin subunits were identified in each ectosome sample together with α4, α5, α6 integrin subunits and MUC18. Other integrin subunits, i.e., α1, β5 and β8, were present in three samples, whereas α9 integrin subunit was found only in ectosomes derived from metastatic WM266-4 cells.

Dissemination of cancer cells is also highly dependent on the activity of matrix metalloproteinases (MMPs). CM ectosomes have already been shown to facilitate the transfer of MMP-1, MMP-2 and MMP-9 and their endogenous activator CD147 [6,7,8,10]. In the present study, the number of proteins involved in matrix disassembly was higher in ectosomes derived from primary CM cells; however, MMP-14 was the only MMP identified in each sample. In contrast, CD147 was present in all samples, suggesting that CM ectosomes may not always transfer MMPs, but have a regulatory role towards the activity of MMPs that are already present.

Noticeably, several markers of epithelial–mesenchymal transition (EMT) were present in CM ectosomes including N-cadherin and vimentin. Overexpression of N-cadherin in primary melanomas and the loss of E-cadherin expression in primary melanomas and metastatic melanomas correlated with worse overall survival of CM patients [18]. The absence of E-cadherin and the presence of N-cadherin in CM ectosomes may reflect their potential to induce or promote EMT in recipient cells and such properties have already been shown for exosomes released by bladder cancer cells [19] and exosomes from plasma of breast cancer patients [20]. Additionally, Qendro et al. [4] demonstrated that the pattern of vimentin expression in exosomes can help predict tumor aggressiveness in different subtypes of melanoma.

Furthermore, ectosomes facilitate the transfer of proangiogenic factors or up-regulate their expression in endothelial cells [21,22,23]. In the present study, comparable numbers of angiogenesis-related proteins were identified in all ectosome samples, including proteins involved in vascular endothelial growth factor (VEGF)-mediated signaling. Nevertheless, VEGF was not found in any sample and only ectosomes from primary WM793 cells carried VEGF receptor 1 (VEGFR1). In addition, neuropilin 1, another VEGF receptor that integrates proangiogenic signals, was present in ectosomes from metastatic WM1205Lu cells.

Cancer progression is also associated with a prothrombotic state. In the studies by Lima et al. [9], melanoma-derived ectosomes displayed a greater procoagulant activity (resulting from elevated levels of tissue factor (TF)) than melanocyte-derived ectosomes. A more recent study showed that accumulation of pancreatic cancer-derived ectosomes at the site of thrombosis is mediated by αvβ1 and αvβ3 integrins [24], both identified in each ectosome sample in the present study. Although we did not identify TF in any CM ectosome sample, other procoagulant molecules such as urokinase plasminogen activator receptor (uPAR), tissue plasminogen activator (tPA) and plasminogen activator inhibitor type 1 (PAI-1) were present in ectosomes from isogenic primary WM793 and metastatic WM1205Lu cells. In addition, PAI-2 was identified in WM793 ectosomes. Its absence in WM1205Lu ectosomes may reflect the primary origin of WM793 cells, since PAI-2 was previously shown to inhibit uPA activity in less invasive tumors in a mice melanoma model [25].

Moreover, in the present study, ectosomes of metastatic origin had a higher percentage of proteins associated with negative regulation of the apoptotic process, including annexins or programmed cell death protein 10 (PDCD10). Furthermore, T-cadherin (cadherin-13) was identified in WM793 and WM1205Lu ectosome samples. Bosserhoff et al. [26] have already demonstrated in mice that the growth of T-cadherin-positive melanoma tumors was diminished in comparison to T-cadherin-negative control, suggesting that loss of T-cadherin desensitizes melanoma cells to apoptosis.

Tumor-derived EVs are associated with suppression of immune response towards transformed cells, for instance, by inducing chemotaxis of blood leukocytes [27]. In the present study, CM ectosomes carried several molecules involved in leukocyte migration such as L1 cell adhesion molecule (L1CAM) and integrins. In a study by Valenti et al. [28], CM ectosomes inhibited differentiation of monocytes to antigen-presenting dendritic cells and the remaining monocytes released transforming growth factor β (TGF-β), which inhibited T-cell cytolytic activity. CM ectosomes were also shown to suppress the immune response by vesicle-associated Fas (FasL) and TRAIL ligands [11]. Our CM ectosome samples did not contain any of the aforementioned molecules; however, multiple proteins involved in T-cell response were identified, including components of major histocompatibility complex I (MHC I). Removal of MHC I molecules via ectosomes may lead to the loss of tumor recognition by cytotoxic T-cells, and may limit the efficacy of cancer immunotherapy.

Cancer cells have also been shown to overexpress different transporter proteins involved in multidrug resistance (MDR) and the efflux of anticancer drugs, i.e., P-glycoprotein (Pgp), multidrug resistance-associated protein 1 (MRP1) and breast cancer resistance protein (BCRP) [29]. In the present study, MRP1 was present in ectosomes released by three CM cell lines (besides WM793) and previous studies by Walsh et al. [30] showed an association of higher MRP1 levels in biopsy specimens with aggressiveness and spread of metastatic melanoma.

### 3.2. Melanoma-Derived Ectosomes as a Source of Potential Disease Biomarkers

Over the years, a number of potential markers for CM have been investigated. However, only a few of them have been recommended for clinical practice, such as lactate dehydrogenase (LDH) or S100B protein, but their poor sensitivity and specificity are major limitations for routine use. The serum levels of S100B, which was found in all CM ectosome samples in the present study, is linked to the tumor burden and reflects a clinical stage of CM. It is not, however, a good indicator of treatment response for patients with stage I, II and III CM, but it is still used as a biomarker for monitoring patients with advanced metastatic disease only [31,32]. In the present study, all CM ectosome samples contained also LDH, the elevated serum level of which is used as an independent and highly significant predictor of survival in CM [33].

The melanoma biomarker field recently saw advancements by different proteomic strategies. Findeisen et al. [34] used matrix-assisted laser desorption/ionization time-of-flight mass spectrometry (MALDI–TOF–MS) and showed that serum amyloidal protein (SAA) and c-reactive protein (CRP) could serve as prognostic serum biomarkers for early-stage CM patients. The same technique was applied for comparative analysis of CM specific spots after two-dimensional gel electrophoresis that revealed five potential biomarkers, i.e., eukaryotic elongation factor 2, enolase 1, aldolase A, glyceraldehyde-3-phosphate dehydrogenase and heterogeneous nuclear ribonucleoprotein A2/B1 [35]. A similar study identified six more proteins overexpressed in melanoma cell lines compared to normal melanocytes, i.e., galectin-1, inosine-5′-monophosphate dehydrogenase 2, serine/threonine-protein phosphatase 2A 65 kDa regulatory subunit A α isoform, protein DJ-1, cyclophilin A and cofilin-1 [36].

Stable isotope labeling with amino acids in the cell culture (SILAC) before MS analysis provides further advantages in biomarker-focused proteomic studies. The peptide peaks of the differentially labeled samples can be accurately quantified relative to each other to determine the peptide and protein ratios. Using SILAC, Liu et al. [37] detected differential expression of CUB-domain-containing protein 1 (CDCP1) in plasma membrane proteome of two CM cell lines of low and high metastatic potential. Similar SILAC-based proteomic comparison of primary WM115 and metastatic WM266-4 cell lines indicated changes in cyclophilin A expression related to the disease stage [38]. Finally, the metastatic potential of CM cells was also correlated by proteomic studies with expression levels of annexin 1 [39], nucleophosmine- and hepatoma-derived growth factor (HDGF) [40].

Only five of the aforementioned proteins (SAA, CRP, CDCP1, HDGF and cyclophilin A) have not been detected in CM ectosomes in the present study, thus proving CM ectosomes as a potential biomarker source. Ectosome content may not only become a source of biomarkers of different disease stages, but may also contribute to differential diagnosis between benign melanocytic lesions and CM. However, while discussing the presence/absence of a particular protein in CM ectosomes, it is necessary to acknowledge the limitations of protein identification by shotgun LC–MS/MS. Such proteomic analysis of unseparated, complex protein mixture allows identification of a much higher number of proteins, which are not lost during gel band or spot excision. On the other hand, signals from underrepresented peptides might be lost among the most abundant proteins. The expression level for particular proteins might have been low and not detected in a given sample replicate in the present study, thus the presence of proteins described as being absent in CM ectosomes cannot be completely ruled out.

Finally, it is important to notice that the number of studies on melanoma EVs is still very limited. Melanoma-derived growth regulatory protein (MIA) and S100B protein were detected in exosomes from serum of CM patients and their quantification presented with diagnostic and prognostic potential towards stage IV of CM [41]. MIA and S100B were also identified in ectosomes in the present study, thus the population of larger vesicles seems to have not less potential in terms of biomarker discovery research. More recently, Cresticelli et al. [42] performed proteomic analysis of small and large EVs isolated from melanoma tumors and confirmed that metastatic melanoma tissues contain a mixture of EVs derived from tumor and immune cells. The same group also showed that two mitochondrial inner membrane proteins: cytochrome c oxidase subunit 2 (MT-CO2) and cytochrome c oxidase subunit 6C (COX6c) are enriched in the plasma of melanoma patients as well as in tumor tissues-derived EVs compared to healthy controls [43]. Studies using human tumor tissues are particularly valuable, since they fully reflect the complexity and cellular interactions present within the tumor microenvironment.

Nevertheless, studies based on the proteomic analyses of EVs only create the premise for the large, independent and multicenter clinical trials that are necessary for the validation of any novel biomarkers. Diagnostic and prognostic potential of elevated numbers of ectosomes, or ectosomes bearing certain molecules, depends on the establishment of proper isolation protocols. To gain valid information for clinical practice, optimal concentrations of uncontaminated vesicle populations with maintained native form and function are required. Our results obtained in the present study are promising and suggest that these investigations should be continued in the future for CM, a disease for which effective biomarkers are still lacking.

## 4. Materials and Methods

### 4.1. Materials

RPMI 1640 GlutaMAX™-I medium, fetal bovine serum (FBS), MicroBCA Protein Assay kit, Alamar Blue cell viability reagent, Acclaim PepMap trap (100 C18, 75 μm × 20 mm, 3 μm particle, 100 Å pore size) and analytical (Acclaim PepMap RSLC C18, 75 µm × 500 mm, 2 µm particle, 100 Å pore size) columns were all purchased from Thermo Fisher Scientific (Waltham, MA, USA). Anti-CD63 mouse monoclonal primary antibody (clone RFAC4, cat. CBL553), Lumi-LightPLUS Western Blotting Kit (including anti-mouse IgG-HRP secondary antibody), Trypsin-EDTA solution, penicillin/streptomycin solution, SpeedBeads™ GE45152105050250 and GE65152105050250, and HEPES buffer were obtained from Sigma-Aldrich (St. Louis, MO, USA). Mouse monoclonal primary antibodies for Arf6 (clone 3A-1, cat. sc-7971) and HSP70 (clone C92F3A-5, cat. sc-66048) were purchased from Santa Cruz Biotechnology (Dallas, TX, USA). Trypsin/Lys-C Mix was the product of Promega (Madison, WI, USA). All remaining chemicals were of analytical grade, commercially available.

### 4.2. Cell Lines and Cell Culture Conditions

Four CM cell lines were obtained from the ESTDAB Melanoma Cell Bank (Tübingen, Germany). WM115 (primary) and WM266-4 (metastatic) cell lines originated from the same individual and represented radial/vertical growth phase and lymph node metastasis, respectively [44]. Primary WM793 cell line, representing vertical growth phase [45], and WM1205Lu, a metastatic variant of WM793 cells obtained from lung metastasis, were also used [46]. Cells were maintained in RPMI 1640 medium with GlutaMAX-I, supplemented with 10% FBS, penicillin (100 unit/mL) and streptomycin (100 μg/mL). Cells were grown in monolayers in a 5% CO_2_ atmosphere at 37 °C in a humidified incubator and passaged after reaching approximately 80% confluence.

### 4.3. Isolation of Ectosomes and Assessment of the Purity of the Ectosome Samples

Sub-confluent cells were cultured for 24 h in serum-free media. Conditioned media were collected and subjected to sequential centrifugation steps. After centrifugations at 400× *g* (5 min, 4 °C), 4000× *g* (20 min, 4° C) and 7000× *g* (20 min, 4 °C), remaining cells and cellular debris were pelleted and discarded, whereas supernatants were collected for ectosome isolation. After final centrifugation at 18,000× *g* (20 min, 4 °C), ectosomes were pelleted and resuspended in ice-cold PBS. A more detailed description of a centrifugation protocol and theoretical model with derived equations are included in Supplementary Materials Data 3.

The purity of the obtained samples was verified by transmission electron microscopy (TEM) as previously described [47] as well as by Nanoparticle Tracking Analysis (NTA). NTA measurements were performed on NanoSight LM 10 (Malvern Panalytical) equipped with a 405 nm laser. For NTA analysis, 10 µL of each ectosome sample was diluted to 2 mL with filtered PBS. The measurement time was set at 30 s and five independent records were collected for each sample. Results were analyzed using NTA 3.1. software and calculated according to the dilutions used. The mean results ± SD were presented on graphs.

Additionally, Western blot (WB) analysis of EV markers was performed. For this purpose, for each CM cell line, whole-cell protein extracts (prepared as described in [47]) and ectosome samples (50 μg of proteins according to MicroBCA method) were separated on 10% SDS-PAGE stain-free precast gels in reducing conditions, and transferred to the PVDF membrane. The purity of ectosome samples was assessed with the use of anti-CD63 (1:2000), anti-HSP70 (1:2000) and anti-ARF6 (1:500) antibodies. After 1 h incubation with primary antibodies, anti-mouse IgG-HRP (1:400) was used as a secondary antibody. Markers were detected using chemiluminescent substrates for HRP and ChemiDoc Imaging System (Bio-Rad).

### 4.4. LC–MS/MS Proteomics

Ectosome lysis, sample preparation for mass spectrometric analysis, LC–MS/MS, and analysis of proteomic data were performed as described below and in [48].

#### 4.4.1. Ectosome Lysis

The ectosome pellets were washed three times with PBS and suspended in 50 µL of lysis buffer (100 mM Tris-HCl pH 7.6, 1% SDS). Lysates were sonicated with Bioruptor UCD-200 (Diagenode, Seraing, Belgium) for 20 min at high intensity (320 W, 30 s/30 s on/off). Next, the samples were denatured at 95 °C under strong agitation for 5 min and centrifuged at 20,000× *g* for 10 min at 20 °C. Proteins were precipitated by adding one volume of trichloroacetic acid to four volumes of the sample. After overnight incubation at –20 °C samples were spun at 10,000× *g* for 15 min at 10 °C and washed two times with ice-cold acetone. The pellets were resuspended in 100 µL of 10 mM HEPES pH 8.5.

#### 4.4.2. Sample Preparation for Mass Spectrometric Analysis

The samples were prepared using paramagnetic bead technology based on the Single-Pot Solid-Phase-Enhanced Sample Preparation (SP3) [49]. GE45152105050250 and GE65152105050250 SpeedBeads™ mixed in a ratio 1:1 were used. The proteins were reduced with dithiothreitol, alkylated with iodoacetamide and digested with Trypsin/Lys-C Mix.

#### 4.4.3. Liquid Chromatography and Tandem Mass Spectrometry (LC–MS/MS)

Peptides were analyzed using an UltiMate 3000 RSLCnano System coupled with a Q-Exactive mass spectrometer (Thermo Fisher Scientific) with DPV-550 Digital PicoView nanospray source (New Objective). The sample was loaded onto a trap column (Acclaim PepMap 100 C18, 75 μm × 20 mm, 3 μm particle, 100 Å pore size) in 2% acetonitrile with 0.05% TFA at a flow rate of 5 μL/min and further resolved on an analytical column (Acclaim PepMap RSLC C18, 75 µm × 500 mm, 2 µm particle, 100 Å pore size) with a 90 min gradient from 2% to 40% acetonitrile in 0.05% formic acid at a flow rate of 200 nL/min. The Q-Exactive was operated in a data-dependent mode using the top eight method. Full-scan MS spectra were acquired with a resolution of 70,000 at *m/z* 200 with automatic gain control (AGC target) of 1 × 10^–6^. The MS/MS spectra were acquired with a resolution of 35,000 at *m/z* 200 with an AGC target of 3 × 10^–6^. The maximum ion accumulation times for the full MS and the MS/MS scans were 120 ms and 110 ms, respectively. Peptides were dynamically excluded from fragmentation within 30 s. Biological replicates were measured twice and searched together.

#### 4.4.4. Analysis of Proteomic Data

The RAW files were processed by the Proteome Discoverer platform (v.1.4, Thermo Fisher Scientific) and searched against the SwissProt database with *Homo sapiens* taxonomy restriction (release February 2019, 20418 sequences) using a locally installed MASCOT search engine (v. 2.5.1, Matrix Science). The following parameters were applied: fixed modification, cysteine carbamidomethylation; variable modifications, methionine oxidation and protein N-terminal acetylation; the peptide mass tolerance, 10 ppm; fragment mass tolerance, 20 mmu. Only tryptic peptides with up to one missed cleavage were considered. Target Decoy PSM Validator was applied with the maximum false discovery rate (FDR) for peptides set to 0.01. The raw data were deposited to the ProteomeXchange Consortium via the MassIVE repository with the dataset identifier PXD017366.

### 4.5. Bioinformatic Analysis

Proteins identified by both biological repetitions of ectosome samples and with at least two peptides were chosen manually to create the final protein lists. Venn diagrams, including Vesiclepedia protein overlap, and gene ontology (GO) analysis, with regard to the cellular compartment, molecular function and biological processes were performed with the use of FunRich 2.0 software with protein UniProt (release 2019_11) database as a reference. For each GO term, six categories with the highest statistical significance of protein enrichment within the given category (calculated as −log10(*p*-value)) were presented on graphs. Additionally, the percentage and number of proteins of selected cancer-related categories with *p* value < 0.001 were compared between ectosome samples from primary and metastatic cells. Entire GO data are provided in Supplementary Materials Data 4. Interaction diagrams from Supplementary Materials Data 2 were prepared with the use of https://string-db.org/ Version: 11.0.

### 4.6. Wound Healing Assay

WM115 and WM793 CM cells were cultured to confluence on 6-well plates. Subsequently, the cell-coated surface was scraped with a 200 µL pipette tip and two different doses (30 µg and 60 µg of proteins) of ectosomes were added for 18 h of incubation. Each wound was photographed in 10 separate fields immediately after scraping (0 h) and after 18 h. The average percentage of wound closure was evaluated by multiple measurements of the wound diameter using Zeiss AxioVision Rel.4.8 image analysis software and calculated as follows:

$$\mathrm{wound}\;\mathrm{closure}\;=\frac{\mathrm{initial}\;\mathrm{wound}\;\mathrm{diameter}\;\left(0\;h\right)\;-\;\mathrm{wound}\;\mathrm{diameter}\;\mathrm{after}\;\left(18\;h\right)}{\mathrm{initial}\;\mathrm{wound}\;\mathrm{diameter}\;\left(0\;h\right)}$$

Results were standardized in relation to the untreated control (taken as 1).

### 4.7. Alamar Blue Cell Viability Assay

WM115 and WM793 CM cells were seeded onto 96-well plates at the density of 1 × 10^4^ cells/100 µL. The next day, a serum-free medium was added and cells were incubated with two doses of ectosomes (30 µg and 60 µg of proteins). After 18 h of incubation, 10% of Alamar Blue reagent was added to each well and after 2 h fluorescence intensity was measured at 560/595 nm. Results were standardized in relation to the untreated control (taken as 1).

### 4.8. Statistical Analysis

Three repetitions of Alamar Blue and wound healing assays were performed for each experimental setting. Analysis of variance (one-way ANOVA) and post-hoc Tukey’s test were later performed with the use of Statistica 12 software to test for statistically significant differences with *p* value < 0.05.

## Supplementary Materials

Supplementary materials can be found at ’https://www.mdpi.com/1422-0067/21/8/2934/s1. LC–MS/MS data are available via ProteomeXchange with identifier PXD017366.

## Abbreviations

CM	Cutaneous Melanoma
EV	Extracellular Vesicles
LC–MS/MS	Liquid Chromatography and Mass Spectrometry
NTA	Nanoparticle Tracking Analysis
TEM	Transmission Electron Microscopy

## References

[1] Karimkhani C., Green A.C., Nijsten T., Weinstock M.A., Dellavalle R.P., Naghavi M., Fitzmaurice C. (2017). The global burden of melanoma: Results from the Global Burden of Disease Study 2015. Br. J. Dermatol..

[2] Raposo G., Stoorvogel W. (2013). Extracellular vesicles: Exosomes, microvesicles, and friends. J. Cell. Biol..

[3] Zaborowski M.P., Balaj L., Breakefield X.O., Lai C.P. (2015). Extracellular vesicles: Composition, biological relevance, and methods of study. Bioscience.

[4] Qendro V., Lundgren D.H., Rezaul K., Mahony F., Ferrell N., Bi A., Latifi A., Chowdhury D., Gygi S., Haas W. (2014). Large-scale proteomic characterization of melanoma expressed proteins reveals nestin and vimentin as biomarkers that can potentially distinguish melanoma subtypes. J. Proteome. Res..

[5] Lazar I., Clement E., Ducoux-Petit M., Denat L., Soldan V., Dauvillier S., Balor S., Burlet-Schiltz O., Larue L., Muller C. (2015). Proteome characterization of melanoma exosomes reveals a specific signature for metastatic cell lines. Pigment Cell Melanoma Res..

[6] Liao C.F., Lin S.H., Chen H.C., Tai C.J., Chang C.C., Li L.T., Yeh C.M., Yeh K.T., Chen Y.C., Hsu T.H. (2012). CSE1L, a novel microvesicle membrane protein, mediates Ras-triggered microvesicle generation and metastasis of tumor cells. Mol. Med..

[7] Hatanaka M., Higashi Y., Fukushige T., Baba N., Kawai K., Hashiguchi T., Su J., Zeng W., Chen X., Kanekura T. (2014). Cleaved CD147 shed from the surface of malignant melanoma cells activates MMP2 produced by fibroblasts. Anticancer Res..

[8] Clancy J.W., Sedgwick A., Rosse C., Muralidharan-Chari V., Raposo G., Method M., Chavrier P., D’Souza-Schorey C. (2015). Regulated delivery of molecular cargo to invasive tumour-derived microvesicles. Nature Commun..

[9] Lima L.G., Oliveira A.S., Campos L.C., Bonamino M., Chammas R., Werneck C., Vicente C.P., Barcinski M.A., Petersen L.C., Monteiro R.Q. (2011). Malignant transformation in melanocytes is associated with increased production of procoagulant microvesicles. Thromb. Haemost..

[10] Zhao X.P., Wang M., Song Y., Song K., Yan T.L., Wang L., Liu K., Shang Z.J. (2015). Membrane microvesicles as mediators for melanoma-fibroblasts communication: Roles of the VCAM-1/VLA-4 axis and the ERK1/2 signal pathway. Cancer Lett..

[11] Martínez-Lorenzo M.J., Anel A., Alava M.A., Piñeiro A., Naval J., Lasierra P., Larrad L. (2004). The human melanoma cell line MelJuSo secretes bioactive FasL and APO2L/TRAIL on the surface of microvesicles. Possible contribution to tumor counterattack. Exp. Cell. Res..

[12] Johnson D.B., Estrada M.V., Salgado R., Sanchez V., Doxie D.B., Opalenik S.R., Vilgelm A.E., Feld E., Johnson A.S., Greenplate A.R. (2016). Melanoma-specific MHC-II expression represents a tumour-autonomous phenotype and predicts response to anti-PD-1/PD-L1 therapy. Nature Commun..

[13] Muhsin-Sharafaldine M.R., Saunderson S.C., Dunn A.C., Faed J.M., Kleffmann T., McLellan A.D. (2016). Procoagulant and immunogenic properties of melanoma exosomes, microvesicles and apoptotic vesicles. Oncotarget..

[14] Hsu M.Y., Shih D.T., Meier F.E., Van Belle P., Hsu J.Y., Elder D.E., Buck C.A., Herlyn M. (1998). Adenoviral gene transfer of beta3 integrin subunit induces conversion from radial to vertical growth phase in primary human melanoma. Am. J. Pathol..

[15] Klein C.E., Dressel D., Steinmayer T., Mauch C., Eckes B., Krieg T., Bankert R.B., Weber L. (1991). Integrin alpha 2 beta 1 is upregulated in fibroblasts and highly aggressive melanoma cells in three-dimensional collagen lattices and mediates the reorganization of collagen I fibrils. J. Cell Biol..

[16] Melchiori A., Mortarini R., Carlone S., Marchisio P.C., Anichini A., Noonan D.M., Albini A. (1995). The alpha 3 beta 1 integrin is involved in melanoma cell migration and invasion. Exp. Cell Res..

[17] Hsu M.Y., Meier F.E., Nesbit M., Hsu J.Y., Van Belle P., Elder D.E., Herlyn M. (2000). E-cadherin expression in melanoma cells restores keratinocyte-mediated growth control and down-regulates expression of invasion-related adhesion receptors. Am. J. Pathol..

[18] Yan S., Holderness B.M., Li Z., Seidel G.D., Gui J., Fisher J.L., Ernstoff M.S. (2016). Epithelial-mesenchymal expression phenotype of primary melanoma and matched metastases and relationship with overall survival. Anticancer Res..

[19] Franzen C.A., Blackwell R.H., Todorovic V., Greco K.A., Foreman K.E., Flanigan R.C., Kuo P.C., Gupta G.N. (2015). Urothelial cells undergo epithelial-to-mesenchymal transition after exposure to muscle invasive bladder cancer exosomes. Oncogenesis.

[20] Galindo-Hernandez O., Gonzales-Vazquez C., Cortes-Reynosa P., Reyes-Uribe E., Chavez-Ocaña S., Reyes-Hernandez O., Sierra-Martinez M., Salazar E.P. (2015). Extracellular vesicles from women with breast cancer promote an epithelial-mesenchymal transition-like process in mammary epithelial cells MCF10A. Tumour Biol..

[21] Al-Nedawi K., Meehan B., Kerbel R.S., Allison A.C., Rak J. (2009). Endothelial expression of autocrine VEGF upon the uptake of tumor-derived microvesicles containing oncogenic EGFR. Proc. Natl. Acad. Sci. USA.

[22] Millimaggi D., Mari M., D’Ascenzo S., Carosa E., Jannini E.A., Zucker S., Carta G., Pavan A., Dolo V. (2007). Tumor vesicle-associated CD147 modulates the angiogenic capability of endothelial cells. Neoplasia.

[23] Liu Y., Zhu X.J., Zeng C., Wu P.H., Wang H.X., Chen Z.C., Li Q.B. (2014). Microvesicles secreted from human multiple myeloma cells promote angiogenesis. Acta Pharmacol. Sin..

[24] Mezouar S., Darbousset R., Dignat-George F., Panicot-Dubois L., Dubois C. (2015). Inhibition of platelet activation prevents the P-selectin and integrin-dependent accumulation of cancer cell microparticles and reduces tumor growth and metastasis in vivo. Int. J. Cancer.

[25] Schroder W.A., Major L.D., Le T.T., Gardner J., Sweet M.J., Janciauskiene S., Suhrbier A. (2014). Tumor cell-expressed SerpinB2 is present on microparticles and inhibits metastasis. Cancer Med..

[26] Bosserhoff A.K., Ellmann L., Quast A.S., Eberle J., Boyle G.M., Kuphal S. (2014). Loss of T-cadherin (CDH-13) regulates AKT signaling and desensitizes cells to apoptosis in melanoma. Mol. Carcinog..

[27] Baj-Krzyworzeka M., Weglarczyk K., Mytar B., Szatanek R., Baran J., Zembala M. (2011). Tumour-derived microvesicles contain interleukin-8 and modulate production of chemokines by human monocytes. Anticancer Res..

[28] Valenti R., Huber V., Filipazzi P., Pilla L., Sovena G., Villa A., Corbelli A., Fais S., Parmiani G., Rivoltini L. (2006). Human tumor-released microvesicles promote the differentiation of myeloid cells with transforming growth factor-beta-mediated suppressive activity on T lymphocytes. Cancer Res..

[29] Jorfi S., Ansa-Addo E.A., Kholia S., Stratton S., Valley S., Lange S., Inal J. (2015). Inhibition of microvesiculation sensitizes prostate cancer cells to chemotherapy and reduces docetaxel dose required to limit tumor growth in vivo. Sci. Rep..

[30] Walsh N., Kennedy S., Larkin A.M., Tryfonopoulos D., Eustace A.J., Mahgoub T., Conway C., Oglesby I., Collins D., Ballot J. (2010). Membrane transport proteins in human melanoma: Associations with tumour aggressiveness and metastasis. Br. J. Cancer.

[31] Kruijff S., Hoekstra H.J. (2012). The current status of S-100B as a biomarker in melanoma. Eur. J. Surg. Oncol..

[32] Barak V., Leibovici V., Peretz T., Kalichman I., Lotem M., Merims S. (2015). Assessing response to new treatments and prognosis in melanoma patients, by the biomarker S-100β. Anticancer Res..

[33] Jurisic V., Radenkovic S., Konjevic G. (2015). The Actual Role of LDH as Tumor Marker, Biochemical and Clinical Aspects. Adv. Exp. Med. Biol..

[34] Findeisen P., Zapatka M., Peccerella T., Matzk H., Neumaier M., Schadendorf D., Ugurel S. (2009). Serum amyloid A as a prognostic marker in melanoma identified by proteomic profiling. J. Clin. Oncol..

[35] Suzuki A., Iizuka A., Komiyama M., Takikawa M., Kume A., Tai S., Ohshita C., Kurusu A., Nakamura Y., Yamamoto A. (2010). Identification of melanoma antigens using a Serological Proteome Approach (SERPA). Cancer Genomics Proteomics.

[36] Caputo E., Maiorana L., Vasta V., Pezzino F.M., Sunkara S., Wynne K., Elia G., Marincola F.M., McCubrey J.A., Libra M. (2011). Characterization of human melanoma cell lines and melanocytes by proteome analysis. Cell Cycle.

[37] Liu H., Ong S.E., Badu-Nkansah K., Schindler J., White F.M., Hynes R.O. (2011). CUB-domaincontaining protein 1 (CDCP1) activates Src to promote melanoma metastasis. Proc. Natl. Acad. Sci. USA.

[38] Al-Ghoul M., Brück T.B., Lauer-Fields J.L., Asirvatham V.S., Zapata C., Kerr R.G., Fields G.B. (2008). Comparative proteomic analysis of matched primary and metastatic melanoma cell lines. J. Proteome Res..

[39] Rondepierre F., Bouchon B., Papon J., Bonnet-Duquennoy M., Kintossou R., Moins N., Maublant J., Madelmont J.C., D’Incan M., Degoul F. (2009). Proteomic studies of B16 lines: Involvement of annexin A1 in melanoma dissemination. Biochim. Biophys. Acta.

[40] Bernard K., Litman E., Fitzpatrick J.L., Shellman Y.G., Argast G., Polvinen K., Everett A.D., Fukasawa K., Norris D.A., Ahn N.G. (2003). Functional proteomic analysis of melanoma progression. Cancer Res..

[41] Alegre E., Zubiri L., Perez-Gracia J.L., González-Cao M., Soria L., Martín-Algarra S., González A. (2016). Circulating melanoma exosomes as diagnostic and prognosis biomarkers. Clin. Chim. Acta.

[42] Crescitelli R., Lässer C., Jang S.C., Cvjetkovic A., Malmhäll C., Karimi N., Höög J.L., Johansson I., Fuchs J., Thorsell A. (2020). Subpopulations of extracellular vesicles from human metastatic melanoma tissue identified by quantitative proteomics after optimized isolation. J Extracell Vesicles..

[43] Jang S.C., Crescitelli R., Cvjetkovic A., Belgrano V., Olofsson Bagge R., Sundfeldt K., Ochiya T., Kalluri R., Lötvall J. (2019). Mitochondrial protein enriched extracellular vesicles discovered in human melanoma tissues can be detected in patient plasma. J Extracell Vesicles..

[44] Herlyn M., Balaban G., Bennicelli J., Guerry D., Halaban R., Herlyn D., Elder D.E., Maul G.G., Steplewski Z., Nowell P.C. (1985). Primary melanoma cells of the vertical growth phase: Similarities to metastatic cells. J. Natl. Cancer Inst..

[45] Cornil I., Theodorescu D., Man S., Herlyn M., Jambrosic J., Kerbel R.S. (1991). Fibroblast cell interactions with human melanoma cells affect tumor cell growth as a function of tumor progression. Proc. Natl. Acad. Sci. USA.

[46] Juhasz I., Albelda S.M., Elder D.E., Murphy G.F., Adachi K., Herlyn D., Valyi-Nagy I.T., Herlyn M. (1993). Growth and invasion of human melanomas in human skin grafted to immunodeficient mice. Am. J. Pathol..

[47] Surman M., Hoja-Łukowicz D., Szwed S., Drożdż A., Stępień E., Przybyło M. (2018). Human melanoma-derived ectosomes are enriched with specific glycan epitopes. Life Sci..

[48] Surman M., Hoja-Łukowicz D., Szwed S., Kędracka-Krok S., Jankowska U., Kurtyka M., Drożdż A., Lityńska A., Stępień E., Przybyło M. (2019). An insight into the proteome of uveal melanoma-derived ectosomes reveals the presence of potentially useful biomarkers. Int. J. Mol. Sci..

[49] Hughes C.S., Foehr S., Garfield D.A., Furlong E.E., Steinmetz L.M., Krijgsveld J. (2014). Ultrasensitive proteome analysis using paramagnetic bead technology. Mol. Syst. Biol..

