# Supplementary material for: Mass Spectrometry-Based Proteomic Characterization of Cutaneous Melanoma Ectosomes Reveals the Presence of Cancer-Related Molecules
Source: Int J Mol Sci. 2020 Apr 22;21(8):2934. doi: 10.3390/ijms21082934 (PMC7215915; doi:10.3390/ijms21082934)
Supplement: Supplementary file 1 [file ijms-21-02934-s001.zip › Supplementary Data/Supplementary Data 2.pdf]

Supplementary Data 2 - Interaction diagrams concerning differential proteins identified in ectosomes derived from different cutaneous melanoma cell lines

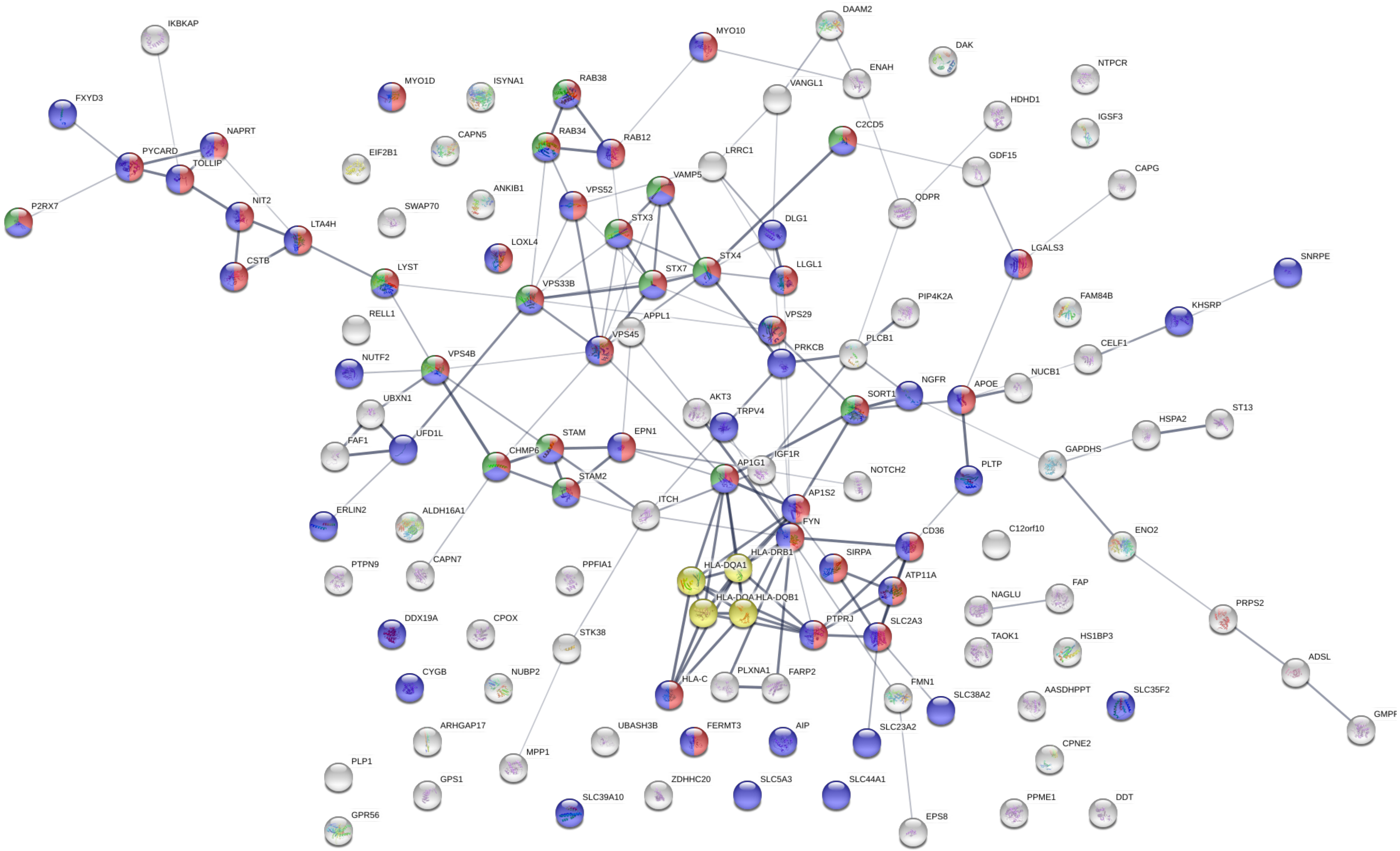

Figure S1

Figure S1 Proteins unique for WM115 ectosomes in comparison to WM973 ectosomes (this means proteins identified in both biological replicates of WM115 ectosomes with at least 2 unique peptides but not found in any WM973 samples). Red colour corresponds to the enriched Gene Ontology term GO:0016192 - vesicle-mediated transport (False Discovery Rate (FDR)=1.16^{-10}, 42 proteins), blue to GO:0006810 - transport (FDR=2.87^{-8}, 62 proteins), green to GO:0016050 - vesicle organisation (FDR=5.46^{-7}, 16 proteins), and yellow to GO:0032395 - MHC class II receptor activity (FDR=0.001, 4 proteins).

PROTEIN ABBREVIATION, protein full name, UNIPROT ID; AASDHPPT - L-aminoadipate-semialdehyde dehydrogenase-phosphopantetheinyl transferase, Q9NRN7; ADSL - Adenylosuccinate lyase, P30566; AIP - AH receptor-interacting protein, O00170; AKT3 - RAC-gamma serine/threonine-protein kinase, Q9Y243; ALDH16A1 - Aldehyde dehydrogenase 16 family member A1 Q8IZ83; ANKIB1 - Ankyrin repeat and IBR domain-containing protein 1, Q9P2G1; AP1G1 - AP-1 complex subunit gamma-1, O43747; AP1S2 - AP-1 complex subunit sigma-2, P56377; APOE - Apolipoprotein E, P02649; APPL1 - DCC-interacting protein 13-alpha, Q9UKG1; ARHGAP17 - Rho GTPase-activating protein 17, Q68EM7; ATP11A - Probable phospholipid-transporting ATPase IH, P98196; C12orf10 - UPF0160 protein MYG1, mitochondrial, Q9HB07; C2CD5 - C2 domain-containing protein 5, Q86YS7; CAPG - Macrophage-capping protein, P40121; CAPN5 - Calpain-5, O15484; CAPN7 - Calpain-7, Q9Y6W3; CD36 - Platelet glycoprotein 4, P16671; CELF1 - CUGBP Elav-like family member 1, Q92879; CHMP6 - Charged multivesicular body protein 6, Q96FZ7; CPNE2 - Copine-2, Q96FN4; CPOX - Oxygen-dependent coproporphyrinogen-III oxidase, mitochondrial, P36551; CSTB - Cystatin-B, P04080; CYGB - Cytoglobin, Q8WWM9; DAAM2 - Dishevelled associated activator of morphogenesis 2, Q86T65; DAK - Triokinase/FMN cyclase, Q3LXA3; DDT - D-dopachrome decarboxylase, P30046; DDX19A - ATP-dependent RNA helicase DDX19A, Q9NUU7; DLG1 - Disks large homolog 1, Q12959; EIF2B1 - Translation initiation factor eIF-2B subunit alpha, Q14232; ENAH - Protein enabled homolog, Q8N8S7; ENO2 - Gamma-enolase, P09104; EPN1 - Epsin-1, Q9Y6I3; EPS8 - Epidermal growth factor receptor kinase substrate 8, Q12929; ERLIN2 - Erlin-2, O94905; FAF1 - FAS-associated factor 1, Q9UNN5; FAM84B - Protein FAM84B, Q96KN1; FAP - Prolyl endopeptidase FAP, Q12884; FARP2 - FERM, ARHGEF and pleckstrin domain-containing protein 2, O94887; FERMT3 - Fermitin family homolog 3, Q86UX7; FMN1 - Formin-1, Q68DA7; FXYD3 - FXYD domain-containing ion transport regulator 3, Q14802; FYN - Tyrosine-protein kinase Fyn, P06241; GAPDHS - Glyceraldehyde-3-phosphate dehydrogenase, testis-specific, O14556; GDF15 - Growth differentiation factor 15, Q99988; GMPR - GMP reductase 1, P36959; GPR56 - Adhesion G-protein coupled receptor G1, Q9Y653; GPS1 - COP9 signalosome complex subunit 1, Q13098; HDHD1 - Pseudouridine-5-phosphatase, Q08623; HLA-C - HLA class I histocompatibility antigen, Cw-7 alpha chain, P10321; HLA-DQA1 - Major histocompatibility complex, class II, DQ alpha 1, P01909; HLA-DQA2 - HLA class II histocompatibility antigen, DQ alpha 2 chain, P01906; HLA-DQB1 - HLA class II histocompatibility antigen, DQ beta 1 chain, P01920; HLA-DRB1 - HLA class II histocompatibility antigen, DRB1-15 beta chain, P13761; HS1BP3 - HCLS1-binding protein 3, Q53T59; HSPA2 - Heat shock-related 70 kDa protein 2, P54652; IGF1R - Insulin-like growth factor 1 receptor, P08069; IGSF3 - Immunoglobulin superfamily member 3, O75054; IKBKAP - Elongator complex protein 1, O95163; ISYNA1 - Inositol-3-phosphate synthase 1, Q9NPH2; ITCH - E3 ubiquitin-protein ligase Itchy homolog, Q96J02; KHSRP - Far upstream element-binding protein 2, Q92945; LGALS3 - Galectin-3, P17931; LLGL1 - Lethal(2) giant larvae protein homolog 1, Q15334; LOXL4 - Lysyl oxidase homolog 4, Q96JB6; LRRC1 - Leucine-rich repeat-containing protein 1, Q9BTT6; LTA4H - Leukotriene A-4 hydrolase, P09960; LYST - Lysosomal-trafficking regulator, Q99698; MPP1 - 55 kDa erythrocyte membrane protein, Q00013; MYO10 - Unconventional myosin-X, Q9HD67; MYO1D - Unconventional myosin-Id, O94832; NAGLU - Alpha-N-acetylglucosaminidase, P54802; NAPRT - Nicotinate phosphoribosyltransferase, Q6XQN6; NGFR - Tumor necrosis factor receptor superfamily member 16, P08138; NIT2 - Omega-amidase NIT2, Q9NQR4; NOTCH2 - Neurogenic locus notch homolog protein 2, Q04721; NTPCR - Cancer-related nucleoside-triphosphatase, Q9BSD7; NUBP2 - Cytosolic Fe-S cluster assembly factor NUBP2, Q9Y5Y2; NUCB1 - Nucleobindin-1, Q02818; NUTF2 - Nuclear transport factor 2, P61970; P2RX7 - P2X purinoceptor 7, Q99572; PIP4K2A - Phosphatidylinositol 5-phosphate 4-kinase type-2 alpha, P48426; PLCB1 - 1-phosphatidylinositol 4,5-bisphosphate phosphodiesterase beta-1, Q9NQ66; PLP1 - Myelin proteolipid protein, P60201; PLTP - Phospholipid transfer protein, P55058; PLXNA1 - Plexin-A1, Q9UIW2; PPFIA1 - Liprin-alpha-1, Q13136; PPME1 - Protein phosphatase methylesterase 1, Q9Y570; PRKCB - Protein kinase C beta type, P05771; PRPS2 - Ribose-phosphate pyrophosphokinase 2, P11908; PTPN9 - Tyrosine-protein phosphatase non-receptor type 9, P43378; PTPRJ - Receptor-type tyrosine-protein phosphatase eta, Q12913; PYCARD - Apoptosis-associated speck-like protein containing a CARD, Q9ULZ3; QDPR - Dihydropteridine reductase, P09417; RAB12 - Ras-related protein Rab-12, Q6IQ22; RAB34 - Ras-related protein Rab-34, Q9BZG1; RAB38 - Ras-related protein Rab-38, P57729; RELL1 - RELT-like protein 1, Q8IUW5; SIRPA - Tyrosine-protein phosphatase non-receptor type substrate 1, P78324; SLC23A2 - Solute carrier family 23 member 2, Q9UGH3; SLC2A3 - Solute carrier family 2, facilitated glucose transporter member 3, P11169; SLC35F2 - Solute carrier family 35 member F2, Q8IXU6; SLC38A2 - Sodium-coupled neutral amino acid transporter 2, Q96QD8; SLC39A10 - Zinc transporter ZIP10, Q9ULF5; SLC44A1 - Choline transporter-like protein 1, Q8WWI5; SLC5A3 - Sodium/myo-inositol cotransporter, P53794; SNRPE - Small nuclear ribonucleoprotein E, P62304; SORT1 - Sortilin, Q99523; ST13 - Hsc70-interacting protein, P50502; STAM - Signal transducing adapter molecule 1, Q92783; STAM2 - Signal transducing adapter molecule 2, O75886; STK38 - Serine/threonine-protein kinase 38, Q15208; STX3 - Syntaxin-3, Q13277; STX4 - Syntaxin-4, Q12846; STX7 - Syntaxin-7, O15400; SWAP70 - Switch-associated protein 70, Q9UH65; TAOK1 - Serine/threonine-protein kinase TAO1, Q7L7X3; TOLLIP - Toll-interacting protein, Q9H0E2; TRPV4 - Transient receptor potential cation channel subfamily V member 4, Q9HBA0; UBASH3B - Ubiquitin-associated and SH3 domain-containing protein B, Q8TF42; UBXN1 - UBX domain-containing protein 1, Q04323; UFD1L - Ubiquitin recognition factor in ER-associated degradation protein 1, Q92890; VAMP5 - Vesicle-associated membrane protein 5, O95183; VANGL1 - VANGL planar cell polarity protein 1 Q8TAA9; VPS29 - Vacuolar protein sorting-associated protein 29, Q9UBQ0; VPS33B - Vacuolar protein sorting-associated protein 33B, Q9H267; VPS45 - Vacuolar protein sorting-associated protein 45, Q9NRW7; VPS4B - Vacuolar protein sorting-associated protein 4B, O75351; VPS52 - Vacuolar protein sorting-associated protein 52 homolog, Q8N1B4; ZDHHC20 - Probable palmitoyltransferase ZDHHC20, Q5W0Z9

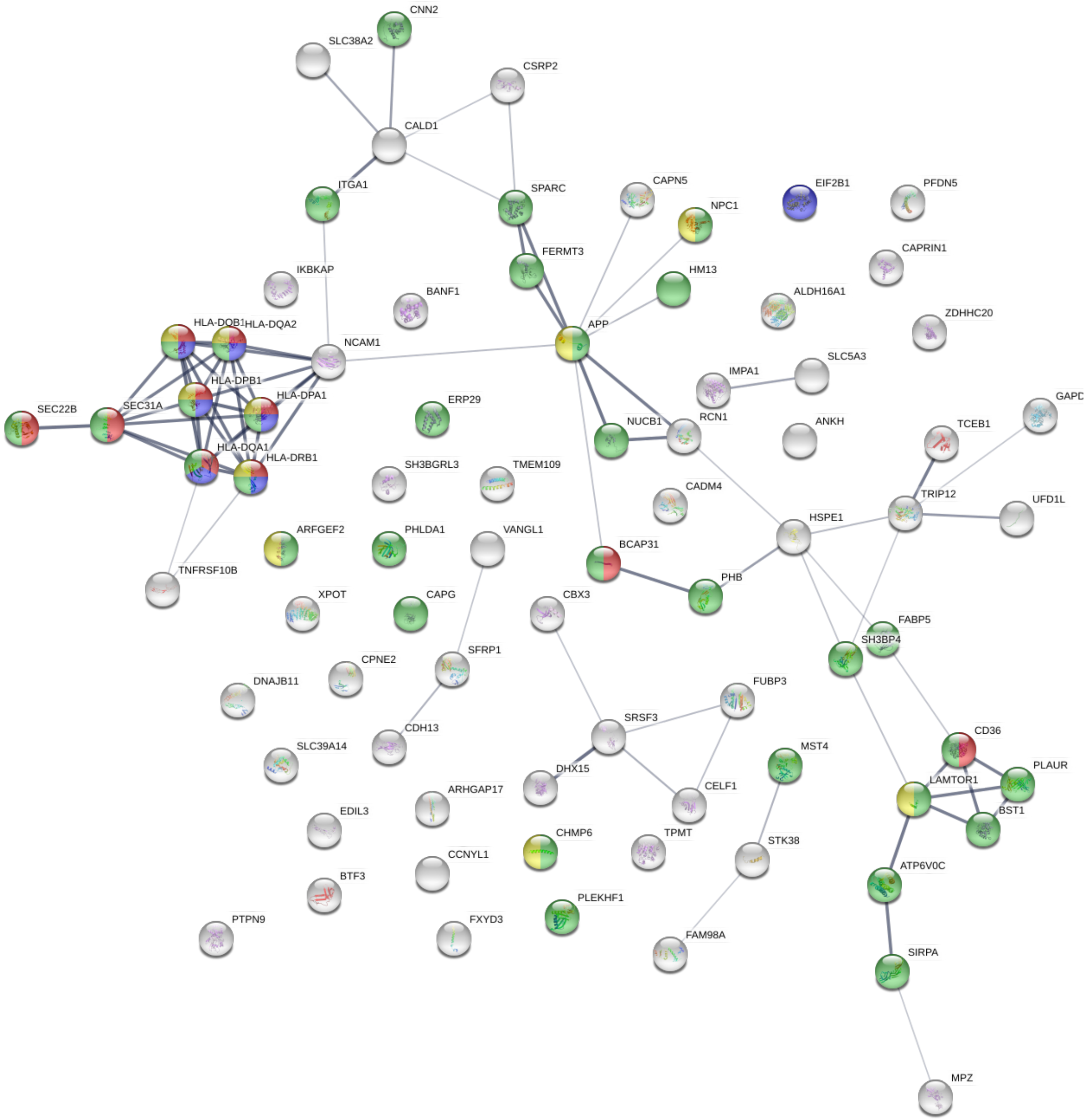
CNN2
SLC38A2
CSRP2
CALD1
ITGA1
SPARC
CAPN5
NPC1
EIF2B1
PFDN5
FERMT3
HM13
CAPRIN1
IKBKAP
BANF1
ALDH16A1
ZDHHC20
HLA-DQB1
HLA-DQA2
APP
NCAM1
SLC5A3
IMPA1
HLA-DPB1
SEC31A
HLA-DPA1
ERP29
GAPD
SEC22B
NUCB1
RCN1
ANKH
TCEB1
HLA-DQA1
HLA-DRB1
SH3BGRL3
TMEM109
CADM4
TRIP12
UFD1L
HSPE1
ARFGEF2
PHLDA1
VANGL1
BCAP31
TNFRSF10B
PHB
XPOT
CAPG
CBX3
FABP5
SH3BP4
SFRP1
CPNE2
FUBP3
DNAJB11
SRSF3
CDH13
CD36
SLC39A14
MST4
DHX15
PLAUR
LAMTOR1
ARHGAP17
CELF1
BST1
EDIL3
CHMP6
TPMT
STK38
CCNYL1
ATP6V0C
BTF3
PLEKHF1
PTPN9
FXYD3
FAM98A
SIRPA
MPZ

Figure S2

Figure S2 Proteins unique for WM115 ectosomes in comparison to WM266 ectosomes (this means proteins identified in both biological replicates of WM115 exosomes with at least 2 unique peptides but not found in any WM266-4 samples). Red colour corresponds to the enriched GO term GO:0048002 - antigen processing and presentation of peptide antigen (FDR=6.86^{-7}, 10 proteins), blue to GO:00050852 - T cell receptor signalling pathway (FDR=6.34^{-5}, 7 proteins), green to GO:0031410 - cytoplasmic vesicle (FDR=7.69^{-10}, 33 proteins), and yellow to GO:00057680 - endosome (FDR=0.0038, 10 proteins).

PROTEIN ABBREVIATION, protein full name, UNIPROT ID; ALDH16A1 - Aldehyde dehydrogenase 16 family member A1 Q8IZ83; ANKH - Progressive ankylosis protein homolog, Q9HCJ1; APP - Amyloid-beta A4 protein, P05067; ARFGEF2 - Brefeldin A-inhibited guanine nucleotide-exchange protein 2, Q9Y6D5; ARHGAP17 - Rho GTPase-activating protein 17, Q68EM7; ATP6V0C - V-type proton ATPase 16 kDa proteolipid subunit, P27449; BANF1 - Barrier-to-autointegration factor, O75531; BCAP31 - B-cell receptor-associated protein 31, P51572; BST1 - ADP-ribosyl cyclase/cyclic ADP-ribose hydrolase 2, Q10588; BTF3 - Transcription factor BTF3, P20290; CADM4 - Cell adhesion molecule 4, Q8NFZ8; CALD1 - Caldesmon, Q05682; CAPG - Macrophage-capping protein, P40121; CAPN5 - Calpain-5, O15484; CAPRIN1 - Caprin-1, Q14444; CBX3 - Chromobox protein homolog 3, Q13185; CCNYL1 - Cyclin-Y-like protein 1, Q8N7R7; CD36 - Platelet glycoprotein 4, P16671; CDH13 - Cadherin-13, P55290; CELF1 - CUGBP Elav-like family member 1, Q92879; CHMP6 - Charged multivesicular body protein 6, Q96FZ7; CNN2 - Calponin-2, Q99439; CPNE2 - Copine-2, Q96FN4; CSRP2 - Cysteine and glycine-rich protein 2, Q16527; DHX15 - Pre-mRNA-splicing factor ATP-dependent RNA helicase DHX15, O43143; DNAJB11 - DnaJ homolog subfamily B member 11, Q9UBS4; EDIL3 - EGF-like repeat and discoidin I-like domain-containing protein 3, O43854; EIF2B1 - Translation initiation factor eIF-2B subunit alpha, Q14232; ERP29 - Endoplasmic reticulum resident protein 29, P30040; FABP5 - Fatty acid-binding protein, epidermal, Q01469; FAM98A - Protein FAM98A, Q8NCA5; FERMT3 - Fermitin family homolog 3, Q86UX7; FUBP3 - Far upstream element-binding protein 3, Q96I24; FXYD3 - FXYD domain-containing ion transport regulator 3, Q14802; GAPDHS - Glyceraldehyde-3-phosphate dehydrogenase, testis-specific, O14556; HLA-DPA1 - HLA class II histocompatibility antigen, DP alpha 1 chain, P20036; HLA-DPB1 - HLA class II histocompatibility antigen, DP beta 1 chain, P04440; HLA-DQA1 - Major histocompatibility complex, class II, DQ alpha 1, P01909; HLA-DQA2 - HLA class II histocompatibility antigen, DQ alpha 2 chain, P01906; HLA-DQB1 - HLA class II histocompatibility antigen, DQ beta 1 chain, P01920; HLA-DRB1 - HLA class II histocompatibility antigen, DRB1-15 beta chain, P20039; HM13 - Histocompatibility minor 13 Q8TCT9; HSPE1 - 10 kDa heat shock protein, mitochondrial, P61604; IKBKAP - Elongator complex protein 1, O95163; IMPA1 - Inositol monophosphatase 1, P29218; ITGA1 - Integrin alpha-1, P56199; LAMTOR1 - Ragulator complex protein LAMTOR1, Q6IAA8; MPZ - Myelin protein P0, P25189; MST4 - Serine/threonine-protein kinase 26, Q9P289; NCAM1 - Neural cell adhesion molecule 1, P13591; NPC1 - Niemann-Pick C1 protein, O15118; NUCB1 - Nucleobindin-1, Q02818; PFDN5 - Prefoldin subunit 5, Q99471; PHB - Prohibitin, P35232; PHLDA1 - Pleckstrin homology-like domain family A member 1, Q8WV24; PLAUR - Urokinase plasminogen activator surface receptor, Q03405; PLEKHF1 - Pleckstrin homology domain-containing family F member 1, Q96S99; PTPN9 - Tyrosine-protein phosphatase non-receptor type 9, P43378; RCN1 - Reticulocalbin-1, Q15293; SEC22B - Vesicle-trafficking protein SEC22b, O75396; SEC31A - Protein transport protein Sec31A, O94979; SFRP1 - Secreted frizzled-related protein 1, Q8N474; SH3BGRL3 - SH3 domain-binding glutamic acid-rich-like protein 3, Q9H299; SH3BP4 - SH3 domain-binding protein 4, Q9P0V3; SIRPA - Tyrosine-protein phosphatase non-receptor type substrate 1, P78324; SLC38A2 - Sodium-coupled neutral amino acid transporter 2, Q96QD8; SLC39A14 - Zinc transporter ZIP14, Q15043; SLC5A3 - Sodium/myo-inositol cotransporter, P53794; SPARC - SPARC, P09486; SRSF3 - Serine/arginine-rich splicing factor 3, P84103; STK38 - Serine/threonine-protein kinase 38, Q15208; TCEB1 - Elongin-C, Q15369; TMEM109 - Transmembrane protein 109, Q9BVC6; TNFRSF10B - Tumor necrosis factor receptor superfamily member 10B, O14763; TPMT - Thiopurine S-methyltransferase, P51580; TRIP12 - E3 ubiquitin-protein ligase TRIP12, Q14669; UFD1L - Ubiquitin recognition factor in ER-associated degradation protein 1, Q92890; VANGL1 - VANGL planar cell polarity protein 1 Q8TAA9; XPOT - Exportin-T, O43592; ZDHHC20 - Probable palmitoyltransferase ZDHHC20, Q5W0Z9

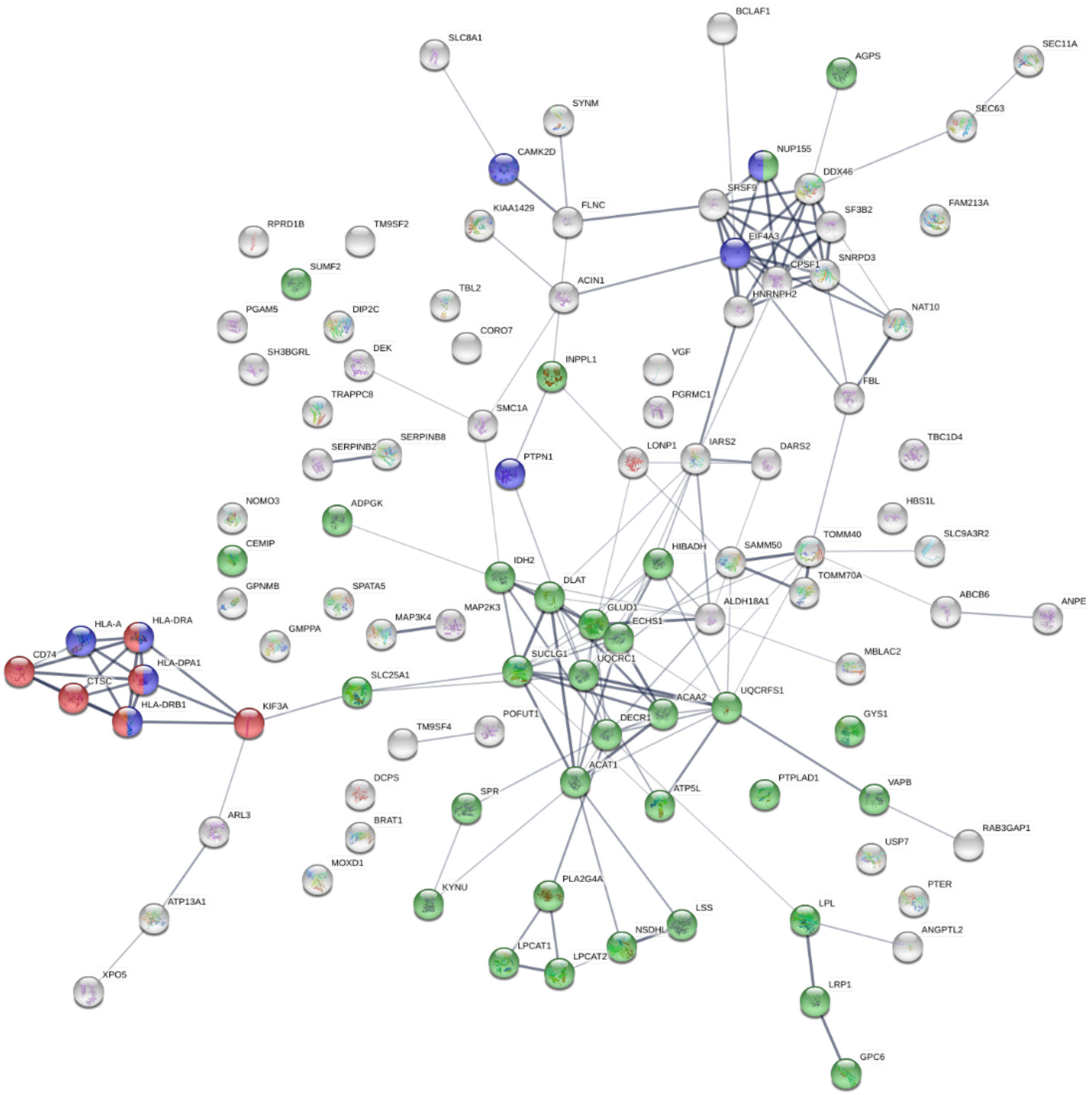
BCLAF1
SLC8A1
AGPS
SEC11A
SYNM
SEC63
CAMK2D
NUP155
DDX46
SRSF9
FLNC
KIAA1429
SF3B2
FAM213A
RPRD1B
TM9SF2
EIF4A3
CPSF1
SNRPD3
SUMF2
ACIN1
HNRNPH2
TBL2
NAT10
PGAM5
DIP2C
CORO7
SH3BGRL
DEK
INPPL1
VGF
FBL
TRAPPC8
PGRMC1
SMC1A
SERPINB8
SERPINB2
IARS2
DARS2
TBC1D4
PTPN1
LONP1
NOMO3
ADPGK
HBS1L
TOMM40
SLC9A3R2
CEMIP
HIBADH
SAMM50
IDH2
TOMM70A
GPNMB
SPATA5
DLAT
ABCB6
ANPE
MAP2K3
ALDH18A1
GLUD1
MAP3K4
ECHS1
HLA-A
HLA-DRA
GMPPA
CD74
SUCLG1
MBLAC2
HLA-DPA1
UQCRC1
CTSC
SLC25A1
UQCRFS1
ACAA2
HLA-DRB1
KIF3A
POFUT1
DECR1
GYS1
TM9SF4
ACAT1
DCPS
PTPLAD1
VAPB
SPR
ATP5L
ARL3
BRAT1
RAB3GAP1
USP7
MOXD1
PTER
PLA2G4A
KYNU
LSS
ATP13A1
LPL
NSDHL
ANGPTL2
LPCAT1
LPCAT2
LRP1
XPO5
GPC6

Figure S3

Figure S3 Proteins unique for WM793 ectosomes in comparison to WM1205Lu ectsomes (this means proteins identified in both biological replicates of WM973 ectosomes with at least 2 unique peptides but not found in any WM1205Lu samples). Red colour corresponds to the enriched GO term: GO:0032395 – MHC class II antigen presentation (FDR=0.0035, 6 proteins), blue to GO:0060333 - Interferon signaling (FDR=0.0015, 8 proteins), and green to GO:1430728 - metabolism (FDR=4.83^{-6}, 32 proteins).

PROTEIN ABBREVIATION, protein full name, UNIPROT ID; ABCB6 - ATP-binding cassette sub-family B member 6, mitochondrial, Q9NP58; ACAA2 - 3-ketoacyl-CoA thiolase, mitochondrial, P42765; ACAT1 - Acetyl-CoA acetyltransferase, mitochondrial, P24752; ACIN1 - Apoptotic chromatin condensation inducer in the nucleus, Q9UKV3; AGPS - Alkyldihydroxyacetonephosphate synthase, peroxisomal, O00116; ALDH18A1 - Delta-1-pyrroline-5-carboxylate synthase, P54886; ANGPTL2 - Angiopoietin-related protein 2, Q9UKU9; ANPEP - Aminopeptidase N, P15144; ARL3 - ADP-ribosylation factor-like protein 3, P36405; ATP13A1 - Manganese-transporting ATPase 13A1, Q9HD20; ATP5L - ATP synthase subunit g, mitochondrial, O75964; BRAT1 - BRCA1-associated ATM activator 1, Q6PJG6; CAMK2D - Calcium/calmodulin-dependent protein kinase type II subunit delta, Q13557; CD74 - HLA class II histocompatibility antigen gamma chain, P04233; CEMIP - Cell migration-inducing and hyaluronan-binding protein, Q8WUJ3; CORO7 - Coronin-7, P57737; CPSF1 - Cleavage and polyadenylation specificity factor subunit 1, Q10570; CTSC - Dipeptidyl peptidase 1, P53634; DARS2 - Aspartate--tRNA ligase, mitochondrial, Q6PI48; DCPS - m7GpppX diphosphatase, Q96C86; DDX46 - Probable ATP-dependent RNA helicase DDX46, Q7L014; DECR1 - 2,4-dienoyl-CoA reductase, mitochondrial, Q16698; DEK - Protein DEK, P35659; DIP2C - Disco interacting protein 2 homolog C Q9Y2E4; DLAT - Dihydrolipoamide S-acetyltransferase, P10515; ECHS1 - Enoyl-CoA hydratase, mitochondrial, P30084; EIF4A3 - Eukaryotic initiation factor 4A-III, P38919; FAM213A - Redox-regulatory protein FAM213A, Q9BRX8; FBL - rRNA 2-O-methyltransferase fibrillarin, P22087; FLNC - Filamin-C, Q14315; GLUD1 - Glutamate dehydrogenase 1, mitochondrial, P00367; GMPPA - Mannose-1-phosphate guanyltransferase alpha, Q96IJ6; GPC6 - Glypican-6, Q9Y625; GPNMB - Transmembrane glycoprotein NMB, Q14956; GYS1 - Glycogen synthase, muscle, P13807; HBS1L - HBS1-like protein, Q9Y450; HIBADH - 3-hydroxyisobutyrate dehydrogenase, mitochondrial, P31937; HLA-A - HLA class I histocompatibility antigen, A-3 alpha chain, P16190; HLA-DPA1 - HLA class II histocompatibility antigen, DP alpha 1 chain, P20036; HLA-DRA - HLA class II histocompatibility antigen, DR alpha chain, P01903; HLA-DRB1 - HLA class II histocompatibility antigen, DRB1-15 beta chain, P01912; HLA-DRB1 - HLA class II histocompatibility antigen, DRB1-15 beta chain, P20039; HLA-DRB1 - HLA class II histocompatibility antigen, DRB1-15 beta chain, Q30134; HNRNPH2 - Heterogeneous nuclear ribonucleoprotein H2, P55795; IARS2 - Isoleucine--tRNA ligase, mitochondrial, Q9NSE4; IDH2 - Isocitrate dehydrogenase , mitochondrial, P48735; INPPL1 - Phosphatidylinositol 3,4,5-trisphosphate 5-phosphatase 2, O15357; KIAA1429 - Protein virilizer homolog, Q69YN4; KIF3A - Kinesin-like protein KIF3A, Q9Y496; LONP1 - Lon protease homolog, mitochondrial, P36776; LPCAT2 - Lysophosphatidylcholine acyltransferase 2, Q7L5N7; LPL - Lipoprotein lipase, P06858; LRP1 - Prolow-density lipoprotein receptor-related protein 1, Q07954; LSS - Lanosterol synthase, P48449; MAP2K3 - Dual specificity mitogen-activated protein kinase kinase 3, P46734; MAP3K4 - Mitogen-activated protein kinase kinase kinase 4, Q9Y6R4; MBLAC2 - Metallo-beta-lactamase domain containing 2, Q68D91; MOXD1 - Monooxygenase DBH like 1, Q6UVY6; NAT10 - RNA cytidine acetyltransferase, Q9H0A0; NOMO3 - Nodal modulator 3, P69849; NUP155 - Nuclear pore complex protein Nup155, O75694; PGAM5 - Serine/threonine-protein phosphatase PGAM5, mitochondrial, Q96HS1; PGRMC1 - Membrane-associated progesterone receptor component 1, O00264; PLA2G4A - Cytosolic phospholipase A2, P47712; POFUT1 - GDP-fucose protein O-fucosyltransferase 1, Q9H488; PTER - Phosphotriesterase-related protein, Q96BW5; PTPLAD1 - Very-long-chain (3R)-3-hydroxyacyl-CoA dehydratase 3, Q9P035; PTPN1 - Tyrosine-protein phosphatase non-receptor type 1, P18031; Q13242; ENSG00000103426 - Coronin-7,Q15738; SRSF9 - Serine/arginine-rich splicing factor 9,Q16719; NSDHL - Sterol-4-alpha-carboxylate 3-dehydrogenase, decarboxylating,Q8NF37; KYNU - Kynureninase,Q9BRR6; LPCAT1 - Lysophosphatidylcholine acyltransferase 1,Q9NYF8; ADPGK - ADP-dependent glucokinase,Q9Y512; BCLAF1 - Bcl-2-associated transcription factor 1,RAB3GAP1 - Rab3 GTPase-activating protein catalytic subunit, Q15042; RPRD1B - Regulation of nuclear pre-mRNA domain-containing protein 1B, Q9NQG5; SEC11A - Signal peptidase complex catalytic subunit SEC11A, P67812; SEC63 - Translocation protein SEC63 homolog, Q9UGP8; SERPINB2 - Plasminogen activator inhibitor 2, P05120; SERPINB8 - Serpin B8, P50452; SF3B2 - Splicing factor 3B subunit 2, Q13435; SH3BGRL - SH3 domain-binding glutamic acid-rich-like protein, O75368; SLC25A1 - Tricarboxylate transport protein, mitochondrial, P53007; SLC8A1 - Sodium/calcium exchanger 1, P32418; SLC9A3R2 - Na(+)/H(+) exchange regulatory cofactor NHE-RF2, Q15599; SMC1A - Structural maintenance of chromosomes protein 1A, Q14683; SNRPD3 - Small nuclear ribonucleoprotein Sm D3, P62318; SPATA5 - Spermatogenesis-associated protein 5, Q8NB90; SPR - Sepiapterin reductase, P35270; SUCLG1 - Succinate--CoA ligase subunit alpha, mitochondrial, P53597; SUMF2 - Sulfatase-modifying factor 2, Q8NBJ7; SYNM - Synemin, O15061; TBC1D4 - TBC1 domain family member 4, O60343; TBL2 - Transducin beta-like protein 2, Q9Y4P3; TM9SF2 - Transmembrane 9 superfamily member 2, Q99805; TM9SF4 - Transmembrane 9 superfamily member 4, Q92544; TOMM40 - Mitochondrial import receptor subunit TOM40 homolog, O96008; TOMM70A - Mitochondrial import receptor subunit TOM70, O94826; TRAPPC8 - Trafficking protein particle complex subunit 8, Q9Y2L5; UQCRC1 - Cytochrome b-c1 complex subunit 1, mitochondrial, P31930; UQCRFS1 - Cytochrome b-c1 complex subunit Rieske, mitochondrial, P47985; USP7 - Ubiquitin carboxyl-terminal hydrolase 7, Q93009; VAPB - Vesicle-associated membrane protein-associated protein B/C, O95292; VGF - Neurosecretory protein VGF, O15240; XPO5 - Exportin-5, Q9HAV4

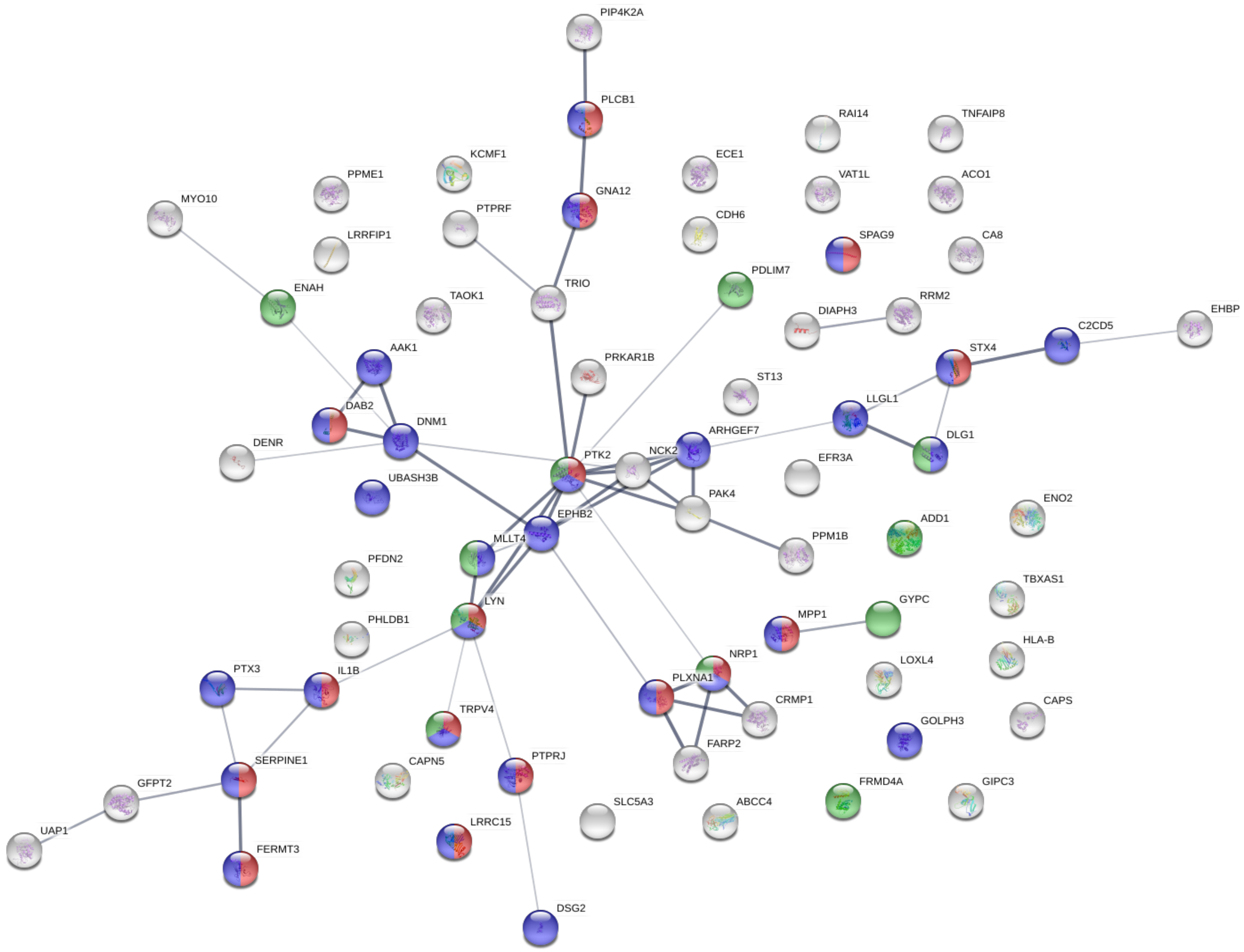
PIP4K2A
PLCB1
RAI14
TNFAIP8
KCMF1
ECE1
PPME1
GNA12
VAT1L
ACO1
MYO10
PTPRF
CDH6
LRRFIP1
SPAG9
CA8
PDLIM7
TRIO
ENAH
TAOK1
DIAPH3
RRM2
EHBP
C2CD5
AAK1
PRKAR1B
STX4
ST13
LLGL1
DAB2
DNM1
ARHGEF7
DLG1
DENR
PTK2
NCK2
EFR3A
UBASH3B
PAK4
ENO2
EPHB2
ADD1
MLLT4
PPM1B
PFDN2
TBXAS1
LYN
GYPC
MPP1
PHLDB1
HLA-B
NRP1
PTX3
IL1B
LOXL4
PLXNA1
CRMP1
CAPS
TRPV4
GOLPH3
SERPINE1
FARP2
PTPRJ
CAPN5
GFPT2
FRMD4A
GIPC3
SLC5A3
ABCC4
UAP1
LRRC15
FERMT3
DSG2

Figure S4

Figure S4 Proteins uniquely found in WM1205Lu ectosomes in comparison to WM973 ectosomes (this means proteins identified in both biological replicates of WM1205Lu ectosomes with at least 2 unique peptides but not found in any WM973 samples). Red colour corresponds to the enriched GO term GO:0030334 **-** regulation of cell migration (FDR=1.45^{-5}, 16 proteins), blue to GO:00032879 **-** regulation of localization (FDR=2.73^{-5}, 28 proteins), and green to GO:0005912 **-** adherens junction (FDR=5.27^{-7}, 11 proteins).

PROTEIN ABBREVIATION, protein full name, UNIPROT ID; AAK1 - AP2-associated protein kinase 1, Q2M2I8; ABCC4 - Multidrug resistance-associated protein 4, O15439; ACO1 - Cytoplasmic aconitate hydratase, P21399; ADD1 - Alpha-adducin, P35611; ARHGEF7 - Rho guanine nucleotide exchange factor 7, Q14155; C2CD5 - C2 domain-containing protein 5, Q86YS7; CA8 - Carbonic anhydrase-related protein, P35219; CAPN5 - Calpain-5, O15484; CAPS - Calcyphosin, Q13938; CDH6 - Cadherin-6, P55285; CRMP1 - Dihydropyrimidinase-related protein 1, Q14194; DAB2 - Disabled homolog 2, P98082; DENR - Density-regulated protein, O43583; DIAPH3 - Protein diaphanous homolog 3, Q9NSV4; DLG1 - Disks large homolog 1, Q12959; DNM1 - Dynamin-1, Q05193; DSG2 - Desmoglein-2, Q14126; ECE1 - Endothelin-converting enzyme 1, P42892; EFR3A - Protein EFR3 homolog A, Q14156; EHBP1 - EH domain-binding protein 1, Q8NDI1; ENAH - Protein enabled homolog, Q8N8S7; ENO2 - Gamma-enolase, P09104; EPHB2 - Ephrin type-B receptor 2, P29323; FARP2 - FERM, ARHGEF and pleckstrin domain-containing protein 2, O94887; FERMT3 - Fermitin family homolog 3, Q86UX7; FRMD4A - FERM domain-containing protein 4A, Q9P2Q2; GFPT2 - Glutamine--fructose-6-phosphate aminotransferase 2, O94808; GIPC3 - PDZ domain-containing protein GIPC3, Q8TF64; GNA12 - Guanine nucleotide-binding protein subunit alpha-12, Q03113; GOLPH3 - Golgi phosphoprotein 3, Q9H4A6; GYPC - Glycophorin-C, P04921; HLA-B - HLA class I histocompatibility antigen, B-7 alpha chain, P30493; IL1B - Interleukin-1 beta, P01584; KCMF1 - E3 ubiquitin-protein ligase KCMF1, Q9P0J7; LLGL1 - Lethal(2) giant larvae protein homolog 1, Q15334; LOXL4 - Lysyl oxidase homolog 4, Q96JB6; LRRC15 - Leucine-rich repeat-containing protein 15, Q8TF66; LRRFIP1 - Leucine-rich repeat flightless-interacting protein 1, Q32MZ4; LYN - Tyrosine-protein kinase Lyn, P07948; MLLT4 - Afadin, P55196; MPP1 - 55 kDa erythrocyte membrane protein, Q00013; MYO10 - Unconventional myosin-X, Q9HD67; NCK2 - Cytoplasmic protein NCK2, O43639; NRP1 - Neuropilin-1, O14786; PAK4 - Serine/threonine-protein kinase PAK 4, O96013; PDLIM7 - PDZ and LIM domain protein 7, Q9NR12; PFDN2 - Prefoldin subunit 2, Q9UHV9; PHLDB1 - Pleckstrin homology like domain family B member 1 Q86UU1; PIP4K2A - Phosphatidylinositol 5-phosphate 4-kinase type-2 alpha, P48426; PLCB1 - 1-phosphatidylinositol 4,5-bisphosphate phosphodiesterase beta-1, Q9NQ66; PLXNA1 - Plexin-A1, Q9UIW2; PPM1B - Protein phosphatase 1B, O75688; PPME1 - Protein phosphatase methylesterase 1, Q9Y570; PRKAR1B - cAMP-dependent protein kinase type I-beta regulatory subunit, P31321; PTK2 - Focal adhesion kinase 1, Q05397; PTPRF - Receptor-type tyrosine-protein phosphatase F, P10586; PTPRJ - Receptor-type tyrosine-protein phosphatase eta, Q12913; PTX3 - Pentraxin-related protein PTX3, P26022; RAI14 - Ankycorbin, Q9P0K7; RRM2 - Ribonucleoside-diphosphate reductase subunit M2, P31350; SERPINE1 - Plasminogen activator inhibitor 1, P05121; SLC5A3 - Sodium/myo-inositol cotransporter, P53794; SPAG9 - C-Jun-amino-terminal kinase-interacting protein 4, O60271; ST13 - Hsc70-interacting protein, P50502; STX4 - Syntaxin-4, Q12846; TAOK1 - Serine/threonine-protein kinase TAO1, Q7L7X3; TBXAS1 - Thromboxane-A synthase, P24557; TNFAIP8 - Tumor necrosis factor alpha-induced protein 8, O95379; TRIO - Triple functional domain protein, O75962; TRPV4 - Transient receptor potential cation channel subfamily V member 4, Q9HBA0; UAP1 - UDP-N-acetylhexosamine pyrophosphorylase, Q16222; UBASH3B - Ubiquitin-associated and SH3 domain-containing protein B, Q8TF42; VAT1L - Synaptic vesicle membrane protein VAT-1 homolog-like, Q9HCJ6

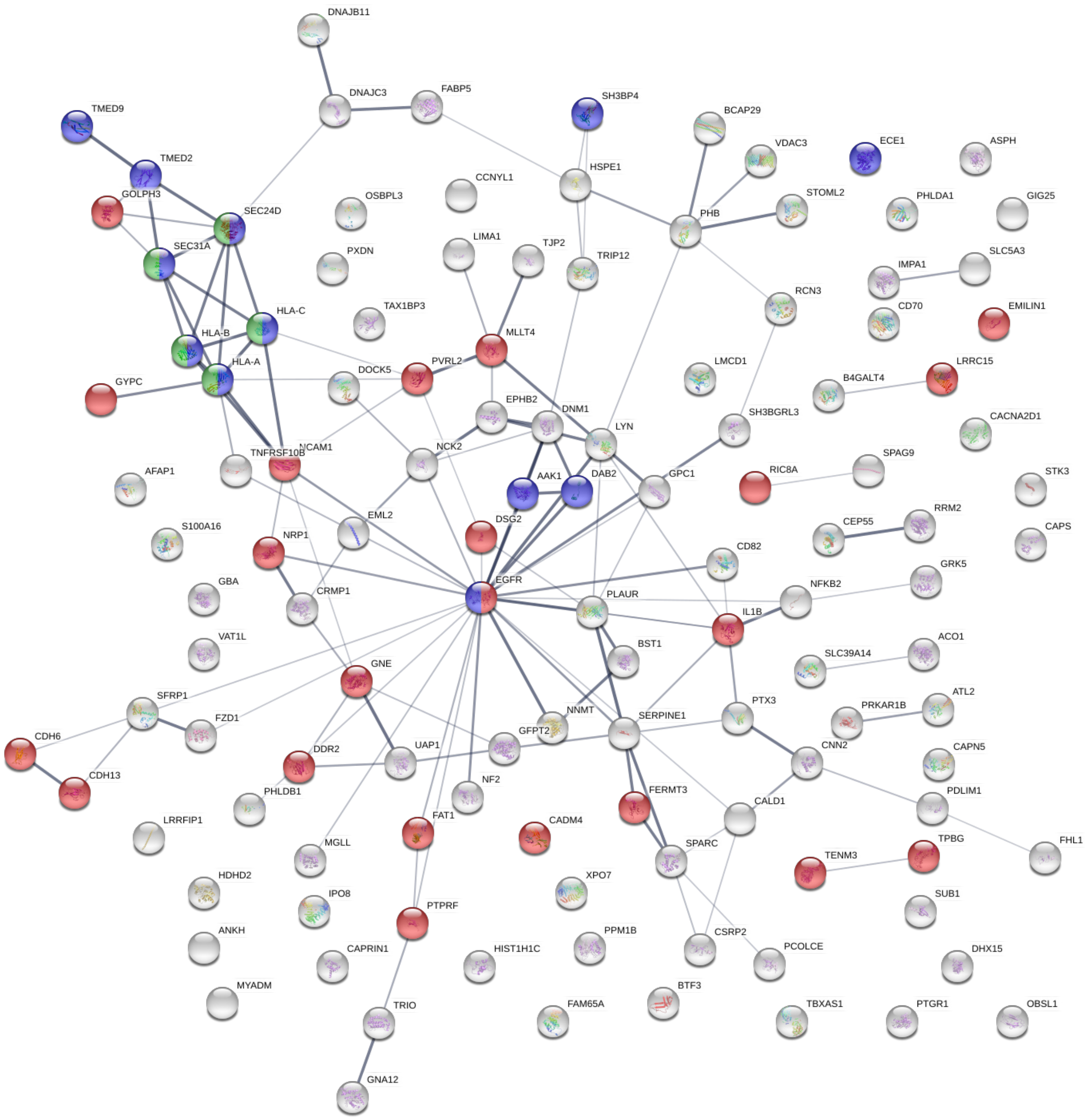
DNAJB11
DNAJC3
FABP5
SH3BP4
BCAP29
TMED9
VDAC3
ECE1
ASPH
TMED2
HSPE1
CCNYL1
GOLPH3
OSBPL3
STOML2
PHLDA1
GIG25
SEC24D
PHB
SEC31A
PXDN
LIMA1
TJP2
TRIP12
IMPA1
SLC5A3
RCN3
HLA-C
TAX1BP3
CD70
EMILIN1
HLA-B
MLLT4
HLA-A
PVRL2
LMCD1
LRRC15
DOCK5
B4GALT4
GYPC
EPHB2
DNM1
SH3BGRL3
CACNA2D1
LYN
NCAM1
TNFRSF10B
NCK2
AFAP1
SPAG9
RIC8A
STK3
AAK1
DAB2
GPC1
EML2
DSG2
RRM2
S100A16
NRP1
CEP55
CAPS
CD82
GBA
EGFR
GRK5
CRMP1
NFKB2
PLAUR
IL1B
VAT1L
ACO1
BST1
SLC39A14
GNE
SFRP1
PTX3
ATL2
FZD1
NNMT
SERPINE1
PRKAR1B
CDH6
GFPT2
UAP1
DDR2
CNN2
CAPN5
CDH13
NF2
PHLDB1
FERMT3
PDLIM1
CALD1
LRRFIP1
FAT1
CADM4
TPBG
MGLL
SPARC
FHL1
TENM3
HDHD2
XPO7
IPO8
SUB1
PTPRF
ANKH
CSRP2
PPM1B
PCOLCE
CAPRIN1
HIST1H1C
DHX15
MYADM
BTF3
TRIO
FAM65A
TBXAS1
PTGR1
OBSL1
GNA12

Figure S5

Figure S5 Proteins unique for WM1205Lu ectosomes in comparison to WM266-4 ectosomes (this means proteins identified in both biological replicates of WM1205Lu ectosomes with at least 2 unique peptides but not found in any WM266-4 samples). Red colour corresponds to the enriched GO term GO:0022610 - biological adhesion (FDR=1.2^{-5}, 22 proteins), blue to GO:0030135 - coated vesicle (FDR=7.93^{-6}, 12 proteins), and green to GO:983170 - antigen presentation: folding, assembly and peptide loading of class I MHC (FDR=0.00047, 5 proteins).

PROTEIN ABBREVIATION, protein full name, UNIPROT ID; DOCK5 - Dedicator of cytokinesis protein 5,AAK1 - AP2-associated protein kinase 1, Q2M2I8; ACO1 - Cytoplasmic aconitate hydratase, P21399; AFAP1 - Actin filament-associated protein 1, Q8N556; ASPH - Aspartyl/asparaginyl beta-hydroxylase, Q12797; ATL2 - Atlastin-2, Q8NHH9; BCAP29 - B-cell receptor-associated protein 29, Q9UHQ4; BST1 - ADP-ribosyl cyclase/cyclic ADP-ribose hydrolase 2, Q10588; BTF3 - Transcription factor BTF3, P20290; CACNA2D1 - Voltage-dependent calcium channel subunit alpha-2/delta-1, P54289; CADM4 - Cell adhesion molecule 4, Q8NFZ8; CALD1 - Caldesmon, Q05682; CAPN5 - Calpain-5, O15484; CAPRIN1 - Caprin-1, Q14444; CCNYL1 - Cyclin-Y-like protein 1, Q8N7R7; CD70 - CD70 antigen, P32970; CD82 - CD82 antigen, P27701; CDH13 - Cadherin-13, P55290; CDH6 - Cadherin-6, P55285; CEP55 - Centrosomal protein of 55 kDa, Q53EZ4; CNN2 - Calponin-2, Q99439; CRMP1 - Dihydropyrimidinase-related protein 1, Q14194; CSRP2 - Cysteine and glycine-rich protein 2, Q16527; DAB2 - Disabled homolog 2, P98082; DDR2 - Discoidin domain-containing receptor 2, Q16832; DHX15 - Pre-mRNA-splicing factor ATP-dependent RNA helicase DHX15, O43143; DNAJB11 - DnaJ homolog subfamily B member 11, Q9UBS4; DNAJC3 - DnaJ homolog subfamily C member 3, Q13217; DNM1 - Dynamin-1, Q05193; DSG2 - Desmoglein-2, Q14126; ECE1 - Endothelin-converting enzyme 1, P42892; EGFR - Epidermal growth factor receptor, P00533; EMILIN1 - EMILIN-1, Q9Y6C2; EML2 - Echinoderm microtubule-associated protein-like 2, O95834; EPHB2 - Ephrin type-B receptor 2, P29323; FABP5 - Fatty acid-binding protein, epidermal, Q01469; FAT1 - Protocadherin Fat 1, Q14517; FERMT3 - Fermitin family homolog 3, Q86UX7; FZD1 - Frizzled-1, Q9UP38; GBA - Glucosylceramidase beta P04062; GFPT2 - Glutamine--fructose-6-phosphate aminotransferase 2, O94808; GIG25 - Serpin peptidase inhibitor, clade A (alpha-1 antiproteinase, antitrypsin), member 3, P01011; GNA12 - Guanine nucleotide-binding protein subunit alpha-12, Q03113; GNE - Bifunctional UDP-N-acetylglucosamine 2-epimerase/N-acetylmannosamine kinase, Q9Y223; GOLPH3 - Golgi phosphoprotein 3, Q9H4A6; GPC1 - Glypican-1, P35052; GRK5 - G protein-coupled receptor kinase 5, P34947; HDHD2 - Haloacid dehalogenase like hydrolase domain containing 2 Q9H0R4; HIST1H1C - Histone H1.2, P16403; HLA-A - HLA class I histocompatibility antigen, A-3 alpha chain, P30443; HLA-B - HLA class I histocompatibility antigen, B-7 alpha chain, P10319; HLA-B - HLA class I histocompatibility antigen, B-7 alpha chain, P30491; HLA-B - HLA class I histocompatibility antigen, B-7 alpha chain, P30493; HLA-C - HLA class I histocompatibility antigen, Cw-7 alpha chain, P30508; HSPE1 - 10 kDa heat shock protein, mitochondrial, P61604; IL1B - Interleukin-1 beta, P01584; IMPA1 - Inositol monophosphatase 1, P29218; IPO8 - Importin-8, O15397; LIMA1 - LIM domain and actin-binding protein 1, Q9UHB6; LMCD1 - LIM and cysteine-rich domains protein 1, Q9NZU5; LRRC15 - Leucine-rich repeat-containing protein 15, Q8TF66; LRRFIP1 - Leucine-rich repeat flightless-interacting protein 1, Q32MZ4; LYN - Tyrosine-protein kinase Lyn, P07948; MGLL - Monoglyceride lipase, Q99685; MYADM - Myeloid associated differentiation marker, Q96S97; NCAM1 - Neural cell adhesion molecule 1, P13591; NCK2 - Cytoplasmic protein NCK2, O43639; NF2 - Merlin, P35240; NFKB2 - Nuclear factor NF-kappa-B p100 subunit, Q00653; NNMT - Nicotinamide N-methyltransferase, P40261; NRP1 - Neuropilin-1, O14786; O14763; CAPS - Calcyphosin,O60513; XPO7 - Exportin-7,O75147; PTX3 - Pentraxin-related protein PTX3,OSBPL3 - Oxysterol-binding protein-related protein 3, Q9H4L5; P04921; B4GALT4 - Beta-1,4-galactosyltransferase 4,P05121; ANKH - Progressive ankylosis protein homolog,P10586; OBSL1 - Obscurin-like protein 1,P26022; SERPINE1 - Plasminogen activator inhibitor 1,P31350; TNFRSF10B - Tumor necrosis factor receptor superfamily member 10B,P55196; GYPC - Glycophorin-C,PCOLCE - Procollagen C-endopeptidase enhancer 1, Q15113; PDLIM1 - PDZ and LIM domain protein 1, O00151; PHB - Prohibitin, P35232; PHLDB1 - Pleckstrin homology like domain family B member 1 Q86UU1; PLAUR - Urokinase plasminogen activator surface receptor, Q03405; PPM1B - Protein phosphatase 1B, O75688; PRKAR1B - cAMP-dependent protein kinase type I-beta regulatory subunit, P31321; PTGR1 - Prostaglandin reductase 1, Q14914; PVRL2 - Nectin-2, Q92692; PXDN - Peroxidasin homolog, Q92626; Q13641; PTPRF - Receptor-type tyrosine-protein phosphatase F,Q13642; MLLT4 - Afadin,Q13938; TPBG - Trophoblast glycoprotein,Q6ZS17; RRM2 - Ribonucleoside-diphosphate reductase subunit M2,Q8WV24; FHL1 - Four and a half LIM domains protein 1,Q9H7D0; PHLDA1 - Pleckstrin homology-like domain family A member 1,Q9HCJ1; AACT - Alpha-1-antichymotrypsin,Q9UIA9; FAM65A - Rho family-interacting cell polarization regulator 1,RCN3 - Reticulocalbin-3, Q96D15; RIC8A - Synembryn-A, Q9NPQ8; S100A16 - Protein S100-A16, Q96FQ6; SEC24D - Protein transport protein Sec24D, O94855; SEC31A - Protein transport protein Sec31A, O94979; SFRP1 - Secreted frizzled-related protein 1, Q8N474; SH3BGRL3 - SH3 domain-binding glutamic acid-rich-like protein 3, Q9H299; SH3BP4 - SH3 domain-binding protein 4, Q9P0V3; SLC39A14 - Zinc transporter ZIP14, Q15043; SLC5A3 - Sodium/myo-inositol cotransporter, P53794; SPAG9 - C-Jun-amino-terminal kinase-interacting protein 4, O60271; SPARC - SPARC, P09486; STK3 - Serine/threonine-protein kinase 3, Q13188; STOML2 - Stomatin-like protein 2, mitochondrial, Q9UJZ1; SUB1 - Activated RNA polymerase II transcriptional coactivator p15, P53999; TAX1BP3 - Tax1-binding protein 3, O14907; TBXAS1 - Thromboxane-A synthase, P24557; TENM3 - Teneurin-3, Q9P273; TJP2 - Tight junction protein ZO-2, Q9UDY2; TMED2 - Transmembrane emp24 domain-containing protein 2, Q15363; TMED9 - Transmembrane emp24 domain-containing protein 9, Q9BVK6; TRIO - Triple functional domain protein, O75962; TRIP12 - E3 ubiquitin-protein ligase TRIP12, Q14669; UAP1 - UDP-N-acetylhexosamine pyrophosphorylase, Q16222; VAT1L - Synaptic vesicle membrane protein VAT-1 homolog-like, Q9HCJ6; VDAC3 - Voltage-dependent anion-selective channel protein 3, Q9Y277

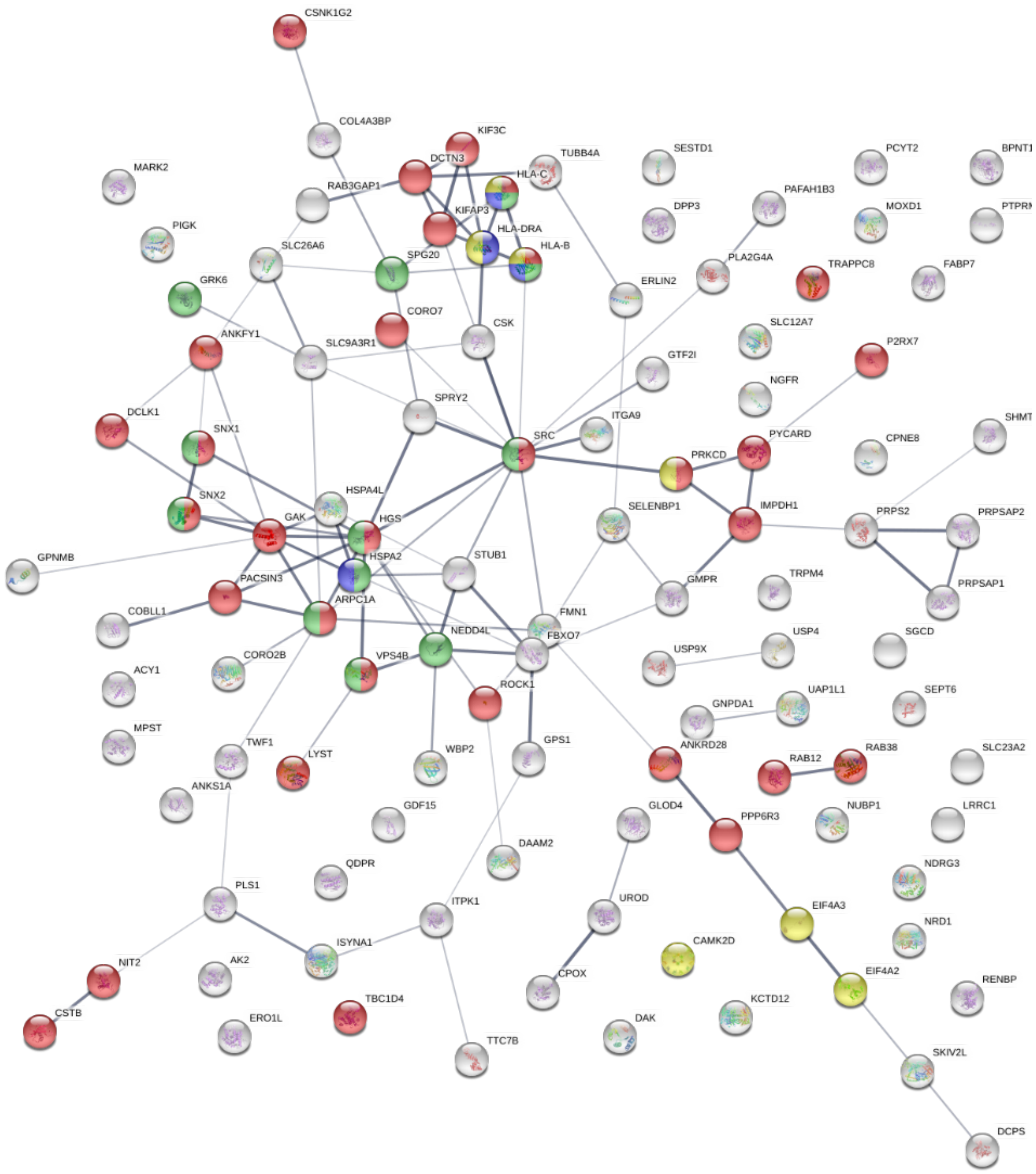
CSNK1G2
COL4A3BP
KIF3C
DCTN3
TUBB4A
SESTD1
PCYT2
BPNT1
MARK2
RAB3GAP1
HLA-C
KIFAP3
DPP3
PAFAH1B3
MOXD1
PTPRN
PIGK
HLA-DRA
HLA-B
SLC26A6
SPG20
PLA2G4A
TRAPPC8
FABP7
GRK6
ERLIN2
CORO7
CSK
SLC12A7
ANKFY1
SLC9A3R1
P2RX7
GTF2I
NGFR
DCLK1
SPRY2
SNX1
ITGA9
SRC
PYCARD
CPNE8
SHMT
PRKCD
HSPA4L
SNX2
IMPDH1
SELENBP1
PRPS2
PRPSAP2
GAK
HGS
GPNMB
HSPA2
STUB1
PACSIN3
TRPM4
ARPC1A
GMPR
PRPSAP1
COBLL1
FMN1
NEDD4L
FBXO7
USP4
SGCD
CORO2B
VPS4B
USP9X
ACY1
ROCK1
UAP1L1
SEPT6
GNPDA1
MPST
TWF1
GPS1
ANKRD28
LYST
WBP2
RAB38
RAB12
SLC23A2
ANKS1A
GDF15
GLOD4
NUBP1
LRRC1
PPP6R3
DAAM2
QDPR
NDRG3
PLS1
ITPK1
UROD
EIF4A3
NRD1
ISYNA1
CAMK2D
AK2
NIT2
CPOX
EIF4A2
RENBP
TBC1D4
KCTD12
CSTB
ERO1L
DAK
TTC7B
SKIV2L
DCPS

Figure S6

Figure S6 Proteins unique for WM266-4 ectosomes in comparison to WM1205Lu ectosomes (this means proteins identified in both biological replicates of WM266-4 ectosomes with at least 2 unique peptides but not found in any WM1205Lu samples). Red colour corresponds to the enriched GO term GO:0016192 - vesicle-mediated transport (FDR=3.96^{-6}, 31 proteins), blue to GO:04612 - antigen processing and presentation (FDR=0.0271, 4 proteins), green to GO:04144 - endocytosis (FDR=3.45^{-6}, 12 proteins), and yellow to GO:913531 - Interferon signaling (FDR=0.0102, 7 proteins).

PROTEIN ABBREVIATION, protein full name, UNIPROT ID; ACY1 - Aminoacylase-1, Q03154; ANKFY1 - Rabankyrin-5, Q9P2R3; ANKS1A - Ankyrin repeat and SAM domain-containing protein 1A, Q92625; ARPC1A - Actin-related protein 2/3 complex subunit 1A, Q92747; BPNT1 - 3(2),5-bisphosphate nucleotidase 1, O95861; CAMK2D - Calcium/calmodulin-dependent protein kinase type II subunit delta, Q13557; COBLL1 - Cordon-bleu WH2 repeat protein like 1 Q53SF7; CORO7 - Coronin-7, P57737; CPOX - Oxygen-dependent coproporphyrinogen-III oxidase, mitochondrial, P36551; CSTB - Cystatin-B, P04080; DCLK1 - Serine/threonine-protein kinase DCLK1, O15075; DPP3 - Dipeptidyl peptidase 3, Q9NY33; EIF4A2 - Eukaryotic initiation factor 4A-II, Q14240; EIF4A3 - Eukaryotic initiation factor 4A-III, P38919; FBXO7 - F-box only protein 7,GAK - Cyclin-G-associated kinase, O14976; GDF15 - Growth differentiation factor 15, Q99988; GLOD4 - Glyoxalase domain containing 4 Q9HC38; GNPDA1 - Glucosamine-6-phosphate isomerase 1, P46926; GPNMB - Transmembrane glycoprotein NMB, Q14956; GPS1 - COP9 signalosome complex subunit 1, Q13098; HLA-B - HLA class I histocompatibility antigen, B-7 alpha chain, P30481; HLA-C - HLA class I histocompatibility antigen, Cw-7 alpha chain, P10321; HLA-DRA - HLA class II histocompatibility antigen, DR alpha chain, P01903; HSPA2 - Heat shock-related 70 kDa protein 2, P54652; HSPA4L - Heat shock 70 kDa protein 4L, O95757; ISYNA1 - Inositol-3-phosphate synthase 1, Q9NPH2; KCTD12 - BTB/POZ domain-containing protein KCTD12, Q96CX2; LRRC1 - Leucine-rich repeat-containing protein 1, Q9BTT6; NDRG3 - Protein NDRG3, Q9UGV2; NEDD4L - E3 ubiquitin-protein ligase NEDD4-like, Q96PU5; NGFR - Tumor necrosis factor receptor superfamily member 16, P08138; NIT2 - Omega-amidase NIT2, Q9NQR4; NRD1 - Nardilysin, O43847; O14782; MARK2 - Serine/threonine-protein kinase MARK2,O14964; COL4A3BP - Collagen type IV alpha-3-binding protein,O15084; RAB3GAP1 - Rab3 GTPase-activating protein catalytic subunit,O15540; HGS - Hepatocyte growth factor-regulated tyrosine kinase substrate,O60256; FABP7 - Fatty acid-binding protein, brain,O75351; PRPSAP2 - Phosphoribosyl pyrophosphate synthase-associated protein 2,O75935; TRAPPC8 - Trafficking protein particle complex subunit 8,O94905; KIF3C - Kinesin-like protein KIF3C,P06132; ERLIN2 - Erlin-2,P13762; DCTN3 - Dynactin subunit 3,P20839; RENBP - N-acylglucosamine 2-epimerase,P25325; - - Sorry, STRING found no proteins by this name in Homo sapiens - -P28827; VPS4B - Vacuolar protein sorting-associated protein 4B,P2RX7 - P2X purinoceptor 7, Q99572; P36959; PTPRM - Receptor-type tyrosine-protein phosphatase mu,P41240; UROD - Uroporphyrinogen decarboxylase,P43250; CSK - Tyrosine-protein kinase CSK,P51606; UAP1L1 - UDP-N-acetylhexosamine pyrophosphorylase-like protein 1,P53384; MPST - 3-mercaptopyruvate sulfurtransferase,P54819; GMPR - GMP reductase 1,P57729; PYCARD - Apoptosis-associated speck-like protein containing a CARD,P78347; ANKRD28 - Serine/threonine-protein phosphatase 6 regulatory ankyrin repeat subunit A,P78368; NUBP1 - Cytosolic Fe-S cluster assembly factor NUBP1,PAFAH1B3 - Platelet-activating factor acetylhydrolase IB subunit gamma, Q15102; PLA2G4A - Cytosolic phospholipase A2, P47712; PLS1 - Plastin-1, Q14651; PRKCD - Protein kinase C delta type, Q05655; PRPS2 - Ribose-phosphate pyrophosphokinase 2, P11908; Q12792; GTF2I - General transcription factor II-I,Q13107; GRK6 - G protein-coupled receptor kinase 6,Q13572; SLC12A7 - Solute carrier family 12 member 7,Q13797; USP9X - Probable ubiquitin carboxyl-terminal hydrolase FAF-X,Q14558; ITGA9 - Integrin alpha-9,Q15042; SLC23A2 - Solute carrier family 23 member 2,Q15477; AK2 - Adenylate kinase 2, mitochondrial,Q3KQV9; PRPSAP1 - Phosphoribosyl pyrophosphate synthase-associated protein 1,Q3LXA3; SKIV2L - Helicase SKI2W,Q5H9R7; USP4 - Ubiquitin carboxyl-terminal hydrolase 4,Q68DA7; IMPDH1 - Inosine-5-monophosphate dehydrogenase 1,Q6UVY6; STUB1 - E3 ubiquitin-protein ligase CHIP,Q7KZI7; TWF1 - Twinfilin-1,Q86T65; TRPM4 - Transient receptor potential cation channel subfamily M member 4,Q86VW0; ENSG00000103426 - Coronin-7,Q86YQ8; SESTD1 - SEC14 domain and spectrin repeat-containing protein 1,Q8N0X7; CPNE8 - Copine-8,Q8TD43; MOXD1 - Monooxygenase DBH like 1,Q92643; CSNK1G2 - Casein kinase I isoform gamma-2,Q92845; DAK - Triokinase/FMN cyclase,Q93008; RAB38 - Ras-related protein Rab-38,Q969T9; PPP6R3 - Serine/threonine-protein phosphatase 6 regulatory subunit 3,Q96C86; WBP2 - WW domain-binding protein 2,Q96HE7; ITPK1 - Inositol-tetrakisphosphate 1-kinase,Q99447; DCPS - m7GpppX diphosphatase,Q99698; PCYT2 - Ethanolamine-phosphate cytidylyltransferase,Q9BXS9; FMN1 - Formin-1,Q9UGH3; DAAM2 - Dishevelled associated activator of morphogenesis 2,Q9UKS6; SPG20 - Spartin,Q9ULZ3; PACSIN3 - Protein kinase C and casein kinase substrate in neurons protein 3,Q9UNE7; PIGK - GPI-anchor transamidase,Q9UQ03; LYST - Lysosomal-trafficking regulator,Q9Y2L5; KIFAP3 - Kinesin-associated protein 3,Q9Y3I1; CORO2B - Coronin-2B,Q9Y5P4; ERO1L - ERO1-like protein alpha,Q9Y666; SLC26A6 - Solute carrier family 26 member 6,QDPR - Dihydropteridine reductase, P09417; RAB12 - Ras-related protein Rab-12, Q6IQ22; ROCK1 - Rho-associated protein kinase 1, Q13464; SELENBP1 - Selenium-binding protein 1, Q13228; SEPT6 - Septin-6, Q14141; SGCD - Delta-sarcoglycan, Q92629; SHMT1 - Serine hydroxymethyltransferase, cytosolic, P34896; SLC9A3R1 - Na(+)/H(+) exchange regulatory cofactor NHE-RF1, O14745; SNX1 - Sorting nexin-1, Q13596; SNX2 - Sorting nexin-2, O60749; SPRY2 - Protein sprouty homolog 2, O43597; SRC - Proto-oncogene tyrosine-protein kinase Src, P12931; TBC1D4 - TBC1 domain family member 4, O60343; TTC7B - Tetratricopeptide repeat protein 7B, Q86TV6; TUBB4A - Tubulin beta-4A chain, P04350
