# Supplementary material for: Mass Spectrometry-Based Proteomic Characterization of Cutaneous Melanoma Ectosomes Reveals the Presence of Cancer-Related Molecules
Source: Int J Mol Sci. 2020 Apr 22;21(8):2934. doi: 10.3390/ijms21082934 (PMC7215915; doi:10.3390/ijms21082934)
Supplement: Supplementary file 1 [file ijms-21-02934-s001.zip › Supplementary Data/Supplementary Data 3.pdf]

**Supplementary Data 3 – Additional information concerning sequential centrifugation protocol**

Our centrifugation protocol comprises 3 pre-preparation steps:

1. at 400 *x g* (5 min, 4ºC)
2. 4,000 *x g* (20 min, 4ºC)
3. 7,000 *x g* (20 min, 4ºC)

to remove remaining cells and cellular debris

and one final centrifugation for ectosome sedimentation at 18,000 *x g* (20 min, 4ºC). This protocol has been designed and optimized after theoretical considerations, based on the model proposed by Livshits et al. (2015) in their paper [1].

During centrifugation at angular velocity $\omega$, three forces act on a mass particle *m*:

1. Centrifugal force: $F = m\omega^2 R$
2. Dynamic friction (Stokes force) $T = -6\pi\eta rcv$
3. Buoyancy force: $W = V\rho_{solv}a$

The condition of force balance $F + W + T = 0$ leads to the following equation (Svedberg equation):

$$v = \frac{dR}{dt} = g_{eff}\frac{d^2}{18\eta}(\rho - \rho_{solv}); \qquad g_{eff} = \omega^2 R \tag{1}$$

where:

| | | | |
|---|---|---|---|
| $m$ | molecular mass | $V$ | molecule volume |
| $\omega$ | angular velocity | $\rho_{solv}$ | solvent density |
| $R$ | distance from the axis of rotation | $a$ | centrifugal acceleration |
| $\eta$ | lepkość dynamiczna medium | $d_S$ | the Stokes diameter (dynamic dimeter), |
| $r_c$ | molecule radius (sphere) | | |
| $v$ | molecule sedimentation velocity | $\rho$ | protein density |

Equation (1) can be written in the following form:

$$\frac{dR}{dt} = \lambda R;\ \lambda = \frac{\omega^2 d^2}{18\eta} \tag{2}$$

Then its solution is as follows

$$R(t) = R(0)e^{\lambda t} \tag{3}$$

Based on the above equation and geometric relationships for the fixed angle A27-8x50 rotor Livshits et al. (2015) determined the "efficiency" parameter of the centrifugation process

*Pelleted(d)* i.e. a fraction of particles of a given diameter *d* which after time *t* travels the distance $L_{sed}$ [1]. We can called this fraction as an enriched fraction.

$$Pelleted(d) = \frac{2}{\pi}\left(arcsin\frac{vt}{L_{sed}} + \frac{vt}{L_{sed}}\sqrt{1-\left(\frac{vt}{L_{sed}}\right)^2}\right) \quad (4)$$

Calculator: http://vesicles.niifhm.ru/index.php?do=1

**Suppl. Table 1.** Centrifugation parameters for the Sorvall LYNX centrifuge (Thermo Scientific) equipped with the fixed angle A27-8x50 rotor (Thermo Scientific).

| | **A27-8x50** |
|---|---|
| $R_{min}$ [mm] | 33 |
| $R_{max}$ [mm] | 107 |
| $\theta$ [º] | 34 |
| tube dimension Ø [mm] | 29 |
| $\rho$ [g/cm$^3$] | 1.1 ÷ 1.3 |
| $\rho_{solv}$ [g/cm$^3$] | 1.0 |
| $\eta$ [cP] | 1.0 ÷ 1.5 |
| *RCF* | 18,000 *x g* |
| *T* [min] | 20, 90 |

$R_{min}$ minimal rotor radius
$R_{max}$ maximal rotor radius
$\theta$ rotor angle
$\rho_{solv}$ solvent density
*RCF* relative centrifugation force [g]
$\eta$ dynamic solvent viscosity
*T* centrifugation time

According to this calculations the enrichment of pellet with soluble/secretory proteins in their average molecular mass around 60kDa (e.g. albumin 63 kDa) is about 0 to 1%, depending on assumed $\rho$ protein density (1.22 to 1.43 g/cm3) [2,3]. If the duration of the centrifugation process is extended to 90 min (more 4 times) protein enrichment will achieve 3% (Suppl. Table 2). Cut off $d_s$ is a molecule (protein) dimension to sediment 100% of protein molecules in those centrifugation conditions.

**Suppl. Table 2.** Calculated protein enrichment in different centrifugation time for typical human plasma proteins.

| $\rho$ [g/cm3] | Cut off $d_s$ | Albumin enrichment [%] | Fibrinogen enrichment [%] |
|---|---|---|---|
| Centrifugation time 20 min | | | |
| 1,22 [2] | 142 | 0 | 3 |
| 1,35 [4] | 113 | 0 | 5 |
| 1,43 [3] | 102 | 1 | 6 |
| Centrifugation time 90 min | | | |
| 1,22 [2] | 67 | 1 | 13 |
| 1,35 [4] | 53 | 2 | 21 |
| 1,43 [3] | 48 | 3 | 25 |

$d_S$ the Stokes diameter (dynamic dimeter), calculated for albumin (7.1 nm) and fibrinogen (21.5 nm) [4]
$\rho$ protein density

1 Livshits M.A., Khomyakova E., Evtushenko E.G., et al. Isolation of exosomes by differential centrifugation: Theoretical analysis of a commonly used protocol [published correction appears in Sci. Rep. 2016;6:21447. Livshits, Mikhail A. [corrected to Livshits, Mikhail A]]. Sci. Rep. 2015;5:17319. Published 2015 Nov 30. doi:10.1038/srep17319

2 Andersson K.M., Hovmoller S. The protein content in crystals and packing coefficients in different space groups. Acta Cryst. 2000. D56, 789-790. doi.org/10.1107/S0907444900005163

3 Quillin M.L., Matthews B.W. Accurate calculation of the density of proteins. Acta Cryst. 2000. D56, 791-794. doi.org/10.1107/S090744490000679X

4 Erickson H.P. Size and shape of protein molecules at the nanometer level determined by sedimentation, gel filtration, and electron microscopy. Biological Procedures Online, 2009. 11, 32-51. doi:10.1007/s12575-009-9008-x
